# Shared genetic architecture of gray matter deficits in schizophrenia and bipolar disorder: evidence from structural neuroimaging–genetic analyses

**DOI:** 10.1017/S0033291726104334

**Published:** 2026-05-14

**Authors:** Yingying Xie, Jiaojiao Du, Yurong Jiang, Yao Zhao, Shiqi Lin, Wenshuang Zhu, Dairong Cao

**Affiliations:** 1 Department of Radiology, The First Affiliated Hospital, Fujian Medical University, Fuzhou, China; 2 Department of Radiology, National Regional Medical Center, Binhai Campus of the First Affiliated Hospital, Fujian Medical University, Fuzhou, China; 3 Department of Breast Imaging, Tianjin Medical University Cancer Institute and Hospital, National Clinical Research Center for Cancer, Tianjin, China; 4 Key Laboratory of Cancer Prevention and Therapy, Tianjin’s Clinical Research Center for Cancer, Key Laboratory of Breast Cancer Prevention and Therapy, Tianjin Medical University, Tianjin, China; 5 Department of Radiology, Tianjin Key Laboratory of Functional Imaging & Tianjin Institute of Radiology, Tianjin Medical University General Hospital, Tianjin, China; 6 Department of Radiology, Xijing Hospital, Fourth Military Medical University, Xi’an, China; 7 Department of Radiology, Beijing Tiantan Hospital, Capital Medical University, Beijing, China; 8 Department of Radiology, Fujian Key Laboratory of Precision Medicine for Cancer, the First Affiliated Hospital, Fujian Medical University, Fuzhou, China; 9 Key Laboratory of Radiation Biology of Fujian Higher Education Institutions, the First Affiliated Hospital, Fujian Medical University, Fuzhou, China

**Keywords:** bipolar disorder, genome-wide association studies, gray matter volume, neurogenetic, schizophrenia

## Abstract

**Background:**

Schizophrenia (SCZ) and bipolar disorder (BD) share substantial clinical and neuroanatomical features, yet the neurogenetic basis underlying their shared gray matter volume (GMV) deficits remains poorly understood.

**Methods:**

We conducted meta-analyses to identify convergent GMV alterations across the two disorders. Genome-wide association studies (GWAS) were performed to uncover genetic variants associated with the shared GMV deficits region in UK Biobank participants. Polygenic risk score (PRS)-GMV associations were analyzed to examine the cumulative influence of genetic risk on GMV in regions with shared deficits. Furthermore, pleiotropic SNPs jointly associated with SCZ, BD, and shared GMV deficits were identified. Spatiotemporal gene expression profiling was utilized to characterize the developmental trajectories, and molecular docking was performed to explore potential drugs.

**Results:**

Meta-analysis revealed consistent overlapping GMV reductions in frontal, temporal, and insular regions across SCZ and BD, based on 6,620 patients and 7,762 controls. GWAS identified 14 SNPs associated with the shared GMV deficits. PRS analyses showed that modestly higher SCZ polygenic risk correlated with decreased GMV of shared regions. Two pleiotropic SNPs – rs11191368 and rs79668541 – were linked to both disorders and the shared GMV deficits. Spatiotemporal expression analyses demonstrated distinct developmental trajectories, and molecular docking highlighted 168 drugs with binding interactions for shared genes.

**Conclusions:**

This study delineates shared neurogenetic mechanisms linking GMV abnormalities to genetic risk across SCZ and BD. Given the cross-sectional design, future longitudinal studies in independent cohorts are warranted to validate these findings and clarify the temporal relationships.

## Introduction

Schizophrenia (SCZ) and bipolar disorder (BD) are highly heritable psychiatric disorders that impose a substantial burden worldwide (Craddock & Sklar, 2013; McGrath, Saha, Chant, & Welham, 2008; Merikangas et al., 2011; Sullivan, Kendler, & Neale, 2003). Individuals with SCZ and BD often confront considerable neuro-cognitive burden, marked risk of functional decline, and enduring structural brain alterations. Despite their separate diagnostic criteria, epidemiological, clinical, and neurobiological overlaps have been documented (Bora & Pantelis, 2015; Craddock & Owen, 2005; Lee et al., 2019). These convergences raise compelling questions about whether SCZ and BD might inhabit overlapping neural and genetic terrain despite their distinct diagnostic boundaries.

Neuroimaging research has robustly documented volumetric reductions in gray matter across both disorders. For example, voxel-based morphometry (VBM) studies consistently show gray matter volume (GMV) reductions in limbic, temporal, and inferior frontal regions in both disorders, with effect sizes that correlate with illness chronicity, antipsychotic exposure, and cognitive impairment (Ellison-Wright & Bullmore, 2010; Farrow et al., 2005; Glahn et al., 2008; Haijma et al., 2013; Kasai et al., 2003; Kempton et al., 2008; Velakoulis et al., 1999). Previous studies have proposed an expanded continuum hypothesis, whereby SCZ and BD share a common psychotic core alongside distinct cognitive and affective cores that are impaired in both groups, and together lie on a shared continuum of neurological abnormalities (Grecucci et al., 2025; Sorella et al., 2019). These observations suggest that GMV impairments may reflect shared neurobiological features across the two disorders. However, the precise voxel-wise patterns of GMV abnormalities that are consistently shared between SCZ and BD, particularly at the meta-analytic level, remain incompletely characterized.

On the other hand, genetic investigations provide complementary evidence for shared liability. Large‐scale genome‐wide association studies (GWAS) and polygenic risk analyses demonstrate substantial genetic correlation between SCZ and BD, implicating common biological pathways. Notably, the previous study reported that genetic risk for SCZ and BD was associated with gray matter volumetric reductions, such as gray matter deficits in frontal, temporal, insular, and thalamic regions (McDonald et al., 2004). These findings are consistent with the hypothesis that volumetric brain alterations may serve as intermediate phenotypes related to genetic liability, although the genetic mechanisms underlying these shared volumetric deficits – and whether they are modulated by the same risk variants common to both illnesses – are yet to be elucidated.

**Figure 1.**
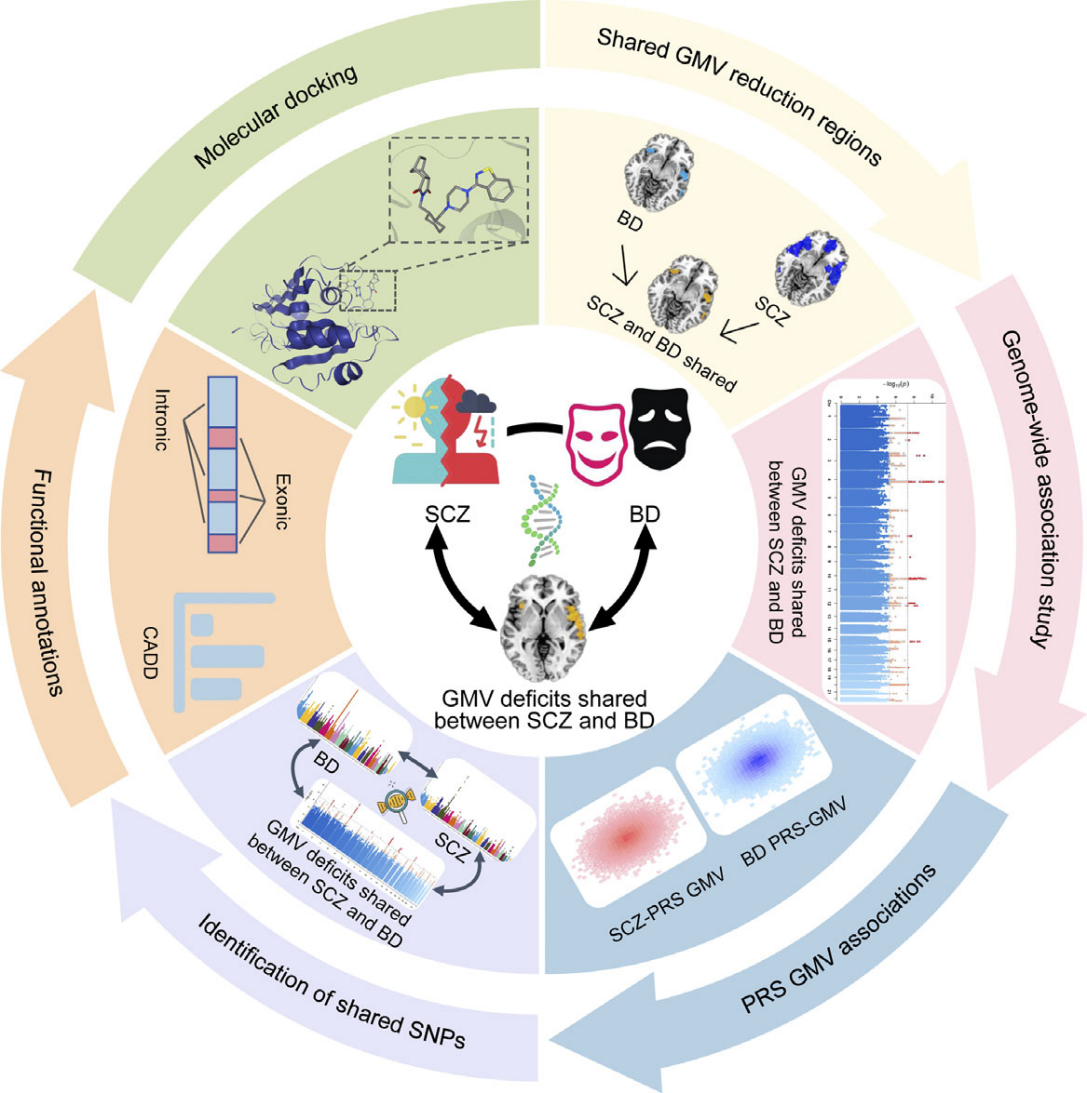
Study design flowchart. This schematic summarizes three sequential components of our investigation: (1) separate voxel-wise meta-analyses of SCZ and BD datasets to delineate regions of overlapping GMV reduction; (2) a genome-wide association study (GWAS) of these shared GMV-deficit regions in the UK Biobank participants, including identification of SNPs and characterization of their developmental expression via the PsychENCODE and Human Brain Transcriptome atlases; (3) associations between BD/SCZ PRS and shared GMV reduction, functional annotation of lead SNPs, and molecular docking of compounds targeting protein products of key shared genes to nominate candidate repurposing agents. Abbreviations: BD, bipolar disorder; CADD, Combined Annotation-Dependent Depletion; GMV, gray matter volume; PRS, polygenic-risk scores; SCZ, schizophrenia.

In this study, we adopted a transdiagnostic framework to investigate shared gray matter volume impairments between SCZ and BD and their genetic correlates. Specifically, we aimed to: (1) identify voxel-wise GMV regions consistently altered across SCZ and BD using large-scale meta-analyses (50 BD datasets; 97 SCZ datasets); (2) examine the genetic architecture underlying these shared GMV deficits through GWAS in a population-based cohort from the UK Biobank; and (3) explore pleiotropic genetic variants and their developmental expression patterns associated with both disorders and the shared imaging phenotype. Secondary analyses further examined associations between shared GMV deficits and polygenic risk scores (PRSs) for SCZ and BD, and explored potential molecular targets through molecular docking analyses. Together, this integrative neuroimaging–genetic–pharmacologic framework characterizes shared patterns of GMV alterations and their genetic correlates across SCZ and BD, providing a systematic resource for investigating transdiagnostic neurobiological mechanisms. The study design flowchart is presented in Figure 1.

## Methods

### Search strategy and selection criteria

This meta-analysis conforms to the Preferred Reporting Items for Systematic Review and Meta-Analysis (PRISMA) guidelines (Moher, Liberati, Tetzlaff, & Altman, 2009). We systematically searched PubMed and Web of Science through May 1, 2025, using the following key terms: (‘bipolar disorder’ OR ‘BD’ OR ‘schizophrenia’ OR ‘schizophrenics’ OR ‘SCZ’) AND (‘voxel-based morphometry’ OR ‘VBM’ OR ‘voxel-based’ OR ‘voxel-wise’) AND (‘gray matter volume’ OR ‘gray matter’ OR ‘grey matter’ OR ‘GMV’ OR ‘GM’). A manual search was also conducted in the reference lists of previous meta-analyses to identify additional studies.

The studies were included if they met all the following criteria: (1) original papers published in peer-reviewed journals; (2) patients with a definitive clinical diagnosis of BD or SCZ; (3) adult subjects; (4) the GMV comparison was conducted between patients with BD or SCZ and HCs; and (5) a whole-brain analysis reported three-dimensional peak coordinates of significant clusters in standard stereotaxic spaces (e.g. Montreal Neurological Institute [MNI] or Talairach). The exclusion criteria were as follows: (1) BD&SCZ patients with comorbid other psychiatric disorders were included; (2) the case–control GMV differences were not compared by a whole-brain voxel-wise analysis; (3) the number of subjects was less than seven in either patient or control groups(Tahmasian et al., 2019); (4) sufficient data (e.g. peak coordinates, *T* or *Z* statistics) could not be obtained from original articles. If the sources of subjects in two studies overlapped, we only included the study with the larger sample size. Moreover, in the case of the longitudinal design, we only included the baseline comparison between patients and controls.

Two researchers independently searched and read the literature, and any disagreements were resolved by consensus. The following information from each included study was extracted, including demographic (e.g. sample size, mean age, and gender) and clinical (illness duration) characteristics, peak coordinates, and statistics (e.g. *T* or *Z* values). The quality of included studies was assessed with a 10-point checklist based on previous studies (Shepherd, Laurens, et al., 2012; Shepherd, Matheson, et al., 2012), including the demographic and clinical characteristics of subjects, methods for image acquisition and analysis, as well as results and conclusions (Shepherd, Laurens, et al., 2012; Shepherd, Matheson, et al., 2012). The score of each item was given as 0, 0.5, or 1 according to whether the criteria were not, partially, or fully met, respectively. The detailed checklist and the scores for each study are shown in Supplementary Tables S1 and S2.

### Voxel-wise meta-analysis

Separate meta-analyses were performed to explore GMV changes in patients with SCZ and BD using the SDM-PSI software (version 6.22, available at https://www.sdmproject.com/) (Albajes-Eizagirre, Solanes, Vieta, & Radua, 2019), SDM-PSI reconstructs effect-size maps from reported peak coordinates and corresponding statistics and implements a random-effects model to account for between-study heterogeneity. Moreover, to evaluate between-study variability in our findings, Cochran’s Q statistic was employed, and the proportion of total variation stemming from heterogeneity was quantified using the I^2^ statistic. Additionally, an assessment of publication bias regarding significant results was conducted through the utilization of Egger’s test. A conjunction analysis was performed by overlapping thresholded meta-analytic maps of GMV changes to examine areas of shared abnormalities across SCZ and BD. Additionally, meta-regression analyses were conducted to explore the effects of clinical factors, such as mean age and illness duration, on GMV changes in patients with SCZ and BD. Conjunction analysis and meta-regression analysis were performed using SDM, with a cluster-level uncorrected *P* < 0.05 threshold and a cluster extent of ≥100 voxels.

### Individual GMV and genetic data from the UK Biobank

The UK Biobank is a population-based cohort drawn from 22 assessment centers across the United Kingdom (Bycroft et al., 2018; Palmer, 2007). Ethical approval for this study was granted by the National Health Service (NHS) Research Ethics Service (Ref. 21/NW/0157), and written informed consent was obtained from all participants. Access to the dataset was approved under application number 75556. Voxel-wise GMV maps were derived from structural MRI data using CAT12 for participants (https://neuro-jena.github.io/cat/). GMV values from brain regions shared between SCZ and BD were then extracted, hereafter referred to as SCZ&BD shared GMV deficits. Specifically, all brain regions showing significant GMV reductions in the SCZ-BD conjunction analysis were combined into a single region of interest (ROI), from which GMV was extracted for each included UK Biobank participant. A conjunction mask was constructed based on regions exhibiting significant GMV deficits in both SCZ and BD identified in the meta-analysis. For each participant, GMV values were extracted voxel-wise within the conjunction mask and summed, yielding a single quantitative GMV phenotype for subsequent GWAS analyses.

The genetic data were obtained from the UK Biobank imputed genotyping dataset. A comprehensive quality control (QC) pipeline was applied. At the sample level, QC excluded non-Caucasian individuals, participants with sex discrepancies, and outliers in heterozygosity or missing genotype rates. Duplicates and relatives were identified by calculating the kinship coefficient using ukbtools, with a threshold of >0.0884 indicating relatedness. Participants with GMV values in the shared brain regions outside the range of the median plus or minus six times the median absolute deviation (MAD) were also excluded. Finally, 35,094 participants were included in the variant-level QC. After converting genetic data from the BGEN format to PLINK binary format, we performed additional standard QC procedures such as minor allele frequency (MAF) ≥ 0.005, imputation quality score (INFO) ≥ 0.6, and Hardy–Weinberg equilibrium (HWE) *P* ≥10^−7^. After these filters, a total of 10,922,046 autosomal SNPs were included in the further analysis.

### GWAS of GMV deficits shared between SCZ and BD

The GWAS was conducted to elucidate the linear relationships between genetic variants and the brain regions with GMV reduction common to SCZ and BD in participants from the UK Biobank. This analysis utilized PLINK 2.0 (available at https://www.cog-genomics.org/plink/2.0/) (Chang et al., 2015; Purcell et al., 2007). Sex, age, age squared, imaging center, total intracranial volume, and the top 10 principal components of genetic ancestry were included as covariates to control for demographic effects, imaging-related confounds, and population stratification. To account for multiple testing across the genome, statistical significance was defined using the conventional genome-wide threshold (*P* < 5 × 10⁻⁸). Independent SNPs were determined through LD clumping using a 250 kb window and an *r^2^* threshold of 0.1, with the 1000 Genomes Project European ancestry dataset serving as the reference panel (Auton et al., 2015).

### PRS-GMV associations for SCZ and BD

PRS was used to assess individuals’ genetic risk for SCZ and BD, incorporating multiple genetic variants across the genome (Martin et al., 2019). Individual standard PRS data for SCZ and BD were derived from the UK Biobank PRS Release (Thompson et al., 2022). Effect sizes of genetic variants in the base dataset were estimated and subsequently used to calculate PRS for each participant in the target dataset using a Bayesian approach (Thompson et al., 2022). Linear regression was performed between PRS and shared GMV regions in SCZ and BD, respectively. Covariates were consistent with those used in the GWAS analysis.

### Identification of shared SNPs

In this study, the Cross-Phenotype Bayesian meta-analysis approach (CPBayes) (Majumdar, Haldar, Bhattacharya, & Witte, 2018) was utilized to identify pleiotropic SNPs shared among BD, SCZ, and BD&SCZ-shared GMV deficits based on GWAS summary statistics. The European (EUR) GWAS summary datasets for SCZ and BD were obtained from the Psychiatric Genomics Consortium (PGC) (O’Connell et al., 2025; Trubetskoy et al., 2022). CPBayes directly estimates posterior probabilities of association for these three phenotypes, highlighting pleiotropic patterns.

CPBayes methods suitable for overlapping samples were employed. To obtain the required input correlation matrix, we implemented the recommended alternative strategy for CPBayes. Briefly, SNPs with univariate association *P* values greater than 0.1 across all traits – indicative of weak or no association with any analyzed phenotype – were initially selected as zero-effect SNPs. An independent set of these zero-effect SNPs was then identified using a linkage disequilibrium (LD) threshold of *r^2^* < 0.01. The correlation matrix of effect sizes (beta) across all traits for the selected independent zero-effect SNPs was subsequently used as the input correlation matrix for CPBayes. CPBayes estimates pleiotropic effects using the local false discovery rate (locFDR), which is computed via Markov Chain Monte Carlo (MCMC) sampling and enables robust estimation of posterior inclusion probabilities across multiple traits. Lower locFDR values indicate stronger evidence that a given SNP exerts pleiotropic effects rather than trait-specific associations. Consistent with prior CPBayes applications (Majumdar et al., 2018; Xie et al., 2024), SNPs with a locFDR of <1 × 10^−4^ were defined as pleiotropic SNPs shared across SCZ, BD, and GMV deficits. Independent SNPs were further defined using a 250 kb window and an *r^2^* threshold of 0.1.

### Functional annotations for pleiotropic SNPs

We used the FUMA (https://fuma.ctglab.nl) (Watanabe, Taskesen, van Bochoven, & Posthuma, 2017) platform to perform systematic functional annotation of SNPs, including those significantly associated with BD&SCZ-shared GMV deficits, and pleiotropic SNPs shared among BD, SCZ, and BD&SCZ-shared GMV deficits. FUMA identified the genomic locations of SNPs (such as intronic, exonic, or intergenic regions) and quantified their potential harm to protein structure and function using the Combined Annotation-Dependent Depletion (CADD) scores. With a threshold of 12.37, the CADD score assessed the pathogenic risk of the SNPs (Rentzsch et al., 2019). In the regulatory element annotation aspect, the regulatory function evidence of SNPs was evaluated through regulomeDB (RDB) scores (Boyle et al., 2012). Lower scores indicate more significant regulatory effects. The minChrState values provide predictions of SNPs’ transcriptional and regulatory effects in different chromatin states (Ernst & Kellis, 2012). The predictions range from 1 to 15 (Watanabe et al., 2017), and 1 to 7 predictions indicate an open chromatin state. In addition, each lead SNP was assigned to its nearest gene based on physical distance in the reference genome, following the default FUMA positional mapping settings. These genes were subsequently used in all downstream functional and enrichment analyses.

### Spatio-temporal gene expression trajectory analysis

To investigate the expression patterns of genes associated with shared GMV deficits in SCZ and BD across distinct life stages in the whole brain, we analyzed data from the PsychENCODE project (Akbarian et al., 2015; Li et al., 2018), which spans a developmental range from 8 post-conception weeks (PCW) to 40 years of age. Nine developmental windows were constructed: 8–9 PCW, 12–13 PCW, 16–17 PCW, 19–22 PCW, 35 PCW to 4 months, 0.5–2.6 postnatal years (PY), 2.8–10.7 PY, 13–19 PY, and 21–40 PY (Li et al., 2018). The expression levels, measured in RPKM (reads per kilobase of transcript per million mapped reads) were used to measure the gene expression level with log2-transformed and normalized. LOESS curves were generated to visualize temporal expression trajectories. For individual gene-level analysis, we utilized data from the Human Brain Transcriptome (HBT) project (https://hbatlas.org/) (Johnson et al., 2009; Kang et al., 2011; Pletikos et al., 2014).

### Molecular docking

Molecular docking, a validated computational structure-based approach, was employed to interrogate ligand–receptor interactions in drug discovery pipelines. In this study, virtual screening was conducted via the DrugRep platform (http://cao.labshare.cn:10180/DrugRep/php/index.php) (Gan et al., 2023), which integrates cavity detection and docking workflows. Potential binding pockets were algorithmically identified using the curvature-based cavity detection approach (CurPocket) (Liu et al., 2020). The topologically largest binding pocket was subjected to structure-based virtual screening using AutoDock Vina (Eberhardt, Santos-Martins, Tillack, & Forli, 2021; Trott & Olson, 2010), with a focus on proteins encoded by the nearest genes of the pleiotropic SNPs shared among BD, SCZ, and BD&SCZ-shared GMV deficits. To prioritize candidate compounds, we included the top 100 docking poses and applied a stringent affinity threshold (Vina score ≤ −7 kcal/mol), a value empirically associated with high-confidence molecular interactions.

## Results

### Included studies and sample characteristics

The search strategy identified 960 studies on BD and 2,321 studies on SCZ. Of these, 47 BD studies (comprising 50 datasets) and 88 SCZ studies (comprising 97 datasets) met the inclusion criteria, encompassing 1,911 BD patients (age = 38.3 ± 6.9 years; 45.0% male) and 2,724 control subjects (age = 36.9 ± 6.5 years; 44.8% male), as well as 4,709 SCZ patients (age = 34.0 ± 8.2 years; 62.6% male) and 5,038 control subjects (age = 33.6 ± 8.1 years; 56.3% male). Sample-size-weighted *t*-tests confirmed no significant age difference between patients and healthy controls for either the BD (*P* = 0.61) or the SCZ (*P* = 0.78) datasets. Similarly, no significant gender difference was found between BD patients and controls (*P* = 0.94). In contrast, a significant gender difference was observed in the SCZ sample (*P* = 0.02), where the proportion of males was higher among patients (62.6%) than among controls (56.3%). Detailed demographic, clinical, and imaging characteristics of the included studies are provided in Supplementary Tables S3 and S4, and the detailed research screening process is presented in Supplementary Figures S1 and S2.

### GMV alterations in SCZ and BD

**Figure 2.**
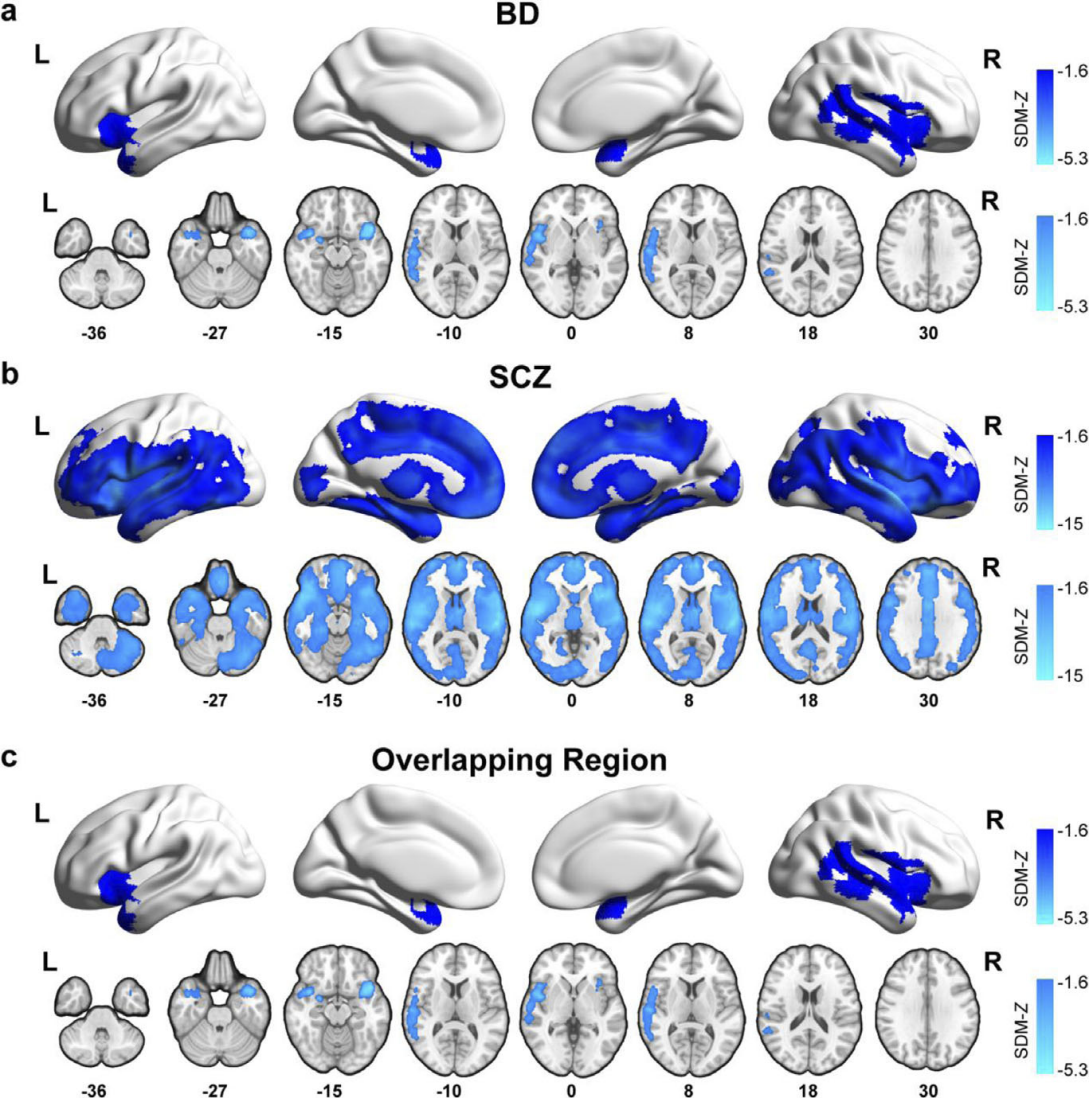
GMV deficits in SCZ and BD. (a,b) The color represents regions showing GMV changes in SCZ and BD identified in the meta-analysis. (c) The color represents regions shared GMV alterations in SCZ and BD patients identified via the conjunction analysis. Abbreviations: BD, bipolar disorder; SCZ, schizophrenia; SDM, seed-based d mapping; GMV, gray matter volume.

Patients with BD mainly showed decreased GMV in the bilateral superior temporal pole (STP), insula, and frontal inferior operculum gyrus (FIOG), right superior temporal gyrus (STG), middle temporal gyrus (MTG), Rolandic operculum, and amygdala (*Z* ≤ −4.536; Figure 2a and Supplementary Table S5). In patients with SCZ, the regions where GMV decreases mainly include bilateral temporal gyrus, frontal gyrus, inferior parietal gyrus (IPG), occipital gyrus, hippocampus, amygdala, fusiform, cingulate, insula, putamen, caudate, and cerebellum (*Z* ≤ −5.316; Figure 2b and Supplementary Table S5). No significantly increased GMV was observed in either disorder. Cochran’s *Q* test revealed no between-study heterogeneity for the significant clusters (all *P* > 0.05 and I^2^ < 30%), and Egger’s test indicated no significant publication bias in the reported results (*P* > 0.05). The shared pattern of GMV reduction in both SCZ and BD is shown in Figure 2c and Supplementary Table S6, mainly distributed in bilateral STP, insula, and FIOG, right STG, and MTG. Additionally, to facilitate direct comparison with prior comprehensive investigations of GMV alterations in SCZ and BD, we summarized key large-scale and meta-analytic studies (Supplementary Table S7) (Fortea et al., 2025; Maggioni et al., 2017; Segal et al., 2023; Sun et al., 2020; Ulugut et al., 2023; Yang et al., 2022). Across these studies, several brain regions – particularly frontal, temporal, and insular cortices – consistently exhibit overlapping GMV reductions in both disorders, which aligns with the common patterns observed in our current results.

### Meta-regression analysis

Meta-regression analyses revealed no significant associations between GMV changes and either age or illness duration in BD. In SCZ, increasing age was negatively associated with GMV in selected cortical and cerebellar regions, including the right cerebellum, right FIOG, right supramarginal gyrus (SMG), left inferior frontal gyrus (IFG), and left cerebellum. In addition, GMV in several regions – such as the right FIOG, bilateral middle occipital gyrus (MOG), bilateral STP, and bilateral cerebellum – showed negative correlations with illness duration. Detailed results are provided in Supplementary Table S8.

### SNPs associated with BD&SCZ-shared GMV deficits

**Figure 3.**
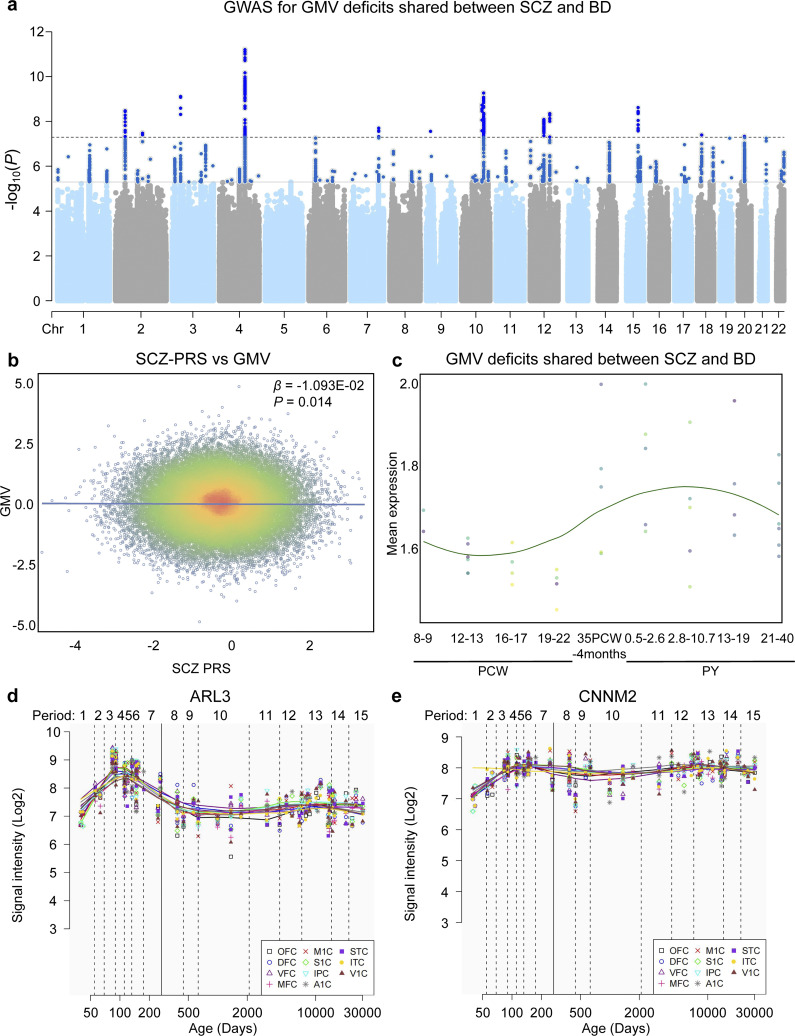
Neurogenetic association and spatio-temporal expression trajectory analysis. (a) Manhattan plots display the GWAS results for GMV deficits shared between SCZ and BD. (b) Scatter plot showing the correlation between SCZ PRS and normalized GMV in shared brain regions. (c) Temporal expression trajectories of genes significantly associated with GMV in shared defective brain regions across the whole brain. (d,e), Spatiotemporal expression patterns of ARL3 and CNNM2. Abbreviations: A1C, primary auditory cortex; BD, bipolar disorder; DFC, dorsolateral prefrontal cortex; IPC, posterior inferior parietal cortex; ITC, inferior temporal cortex; M1C, primary motor cortex; MFC, medial prefrontal cortex; OFC, orbital prefrontal cortex; S1C, primary somatosensory cortex; SCZ, schizophrenia; STC, posterior superior temporal cortex; V1C, primary visual cortex; VFC, ventrolateral prefrontal cortex.

To identify genetic variants associated with brain regions exhibiting GMV reduction shared between SCZ and BD, we performed a GWAS using data from 35,094 participants of the UK Biobank cohort. We identified 14 independent SNPs significantly associated with BD&SCZ-shared GMV deficits (Figure 3a, Supplementary Figure S3 and Supplementary Table S9). The top associated SNP, rs36003774, reached a significance level of 6.25 × 10^−12^ and maps to an intronic region. Notably, two SNPs exhibited high functional impact scores: rs2274224 (exonic) with a CADD score of 17.35 and rs10784447 (intronic) with a CADD score of 20.6, suggesting potential functional consequences. Additionally, 13 SNPs showed a minChrState value of <8.

In addition, to assess whether hemispheric asymmetry influenced our findings, we conducted sensitivity analyses by separately constructing left- and right-hemisphere whole-brain GMV phenotypes using identical preprocessing pipelines and voxel-wise extraction procedures. For each hemisphere, voxel-wise GMV values were extracted and aggregated to generate a single hemispheric GMV measure for each participant. GWAS were then performed independently for left- and right-hemisphere phenotypes, adjusting for the same set of covariates. GWAS results derived from the left and right hemispheres showed high concordance across the genome. To further characterize and compare the underlying polygenic architectures, we subsequently applied MiXeR analyses (https://github.com/precimed/mixer) to both hemispheric phenotypes (Frei et al., 2019), revealing a substantial overlap in polygenic components between the left-hemisphere and right-hemisphere GMV measures, evidenced by high Dice coefficients (Dice = 0.987) and strong shared genetic enrichment (Supplementary Figure S4).

### PRS GMV association analyses

For SCZ PRS, we observed a nominal negative correlation with GMV in the shared regions (β = −1.093 × 10^−2^, *P* = 0.014) (Figure 3b), indicating that higher genetic risk for SCZ is associated with reduced GMV in these commonly affected brain areas. No significant association was found for BD (Supplementary Figure S5).

### Pleiotropic SNPs shared among BD, SCZ, and BD&SCZ-shared GMV deficits

Two genome-wide significant pleiotropic SNPs were identified across three phenotypes (Supplementary Table S10). The first, rs11191368, maps to an intronic region of *ARL3* (ADP-ribosylation factor-like 3) and is characterized by a CADD score of 5.017, an RDB score of 7, and a minChrState value of 4. The second, rs79668541, is located in an intronic region of *CNNM2* (cyclin and CBS domain divalent metal cation transport mediator 2), with an RDB score of 6 and a minChrState value of 5.

### Spatio-temporal gene expression trajectory analysis

For genes associated with BD&SCZ-shared GMV deficits, the temporal expression trajectory across the whole brain exhibited a dynamic pattern. As illustrated by the LOESS-smoothed curve, mean expression levels showed a trough around 12–13 PCW, followed by a progressive increase that peaked during the 2.8–10.7 PY window, and then a gradual decline into adulthood (21–40 PY) (Figure 3c). Using the HBT dataset, we further examined the spatiotemporal expression dynamics of *ARL3* and *CNNM2*, two genes shared among SCZ, BD, and the overlapping GMV deficits. For *ARL3*, signal intensity increased between 50 and 100 days post-conception across most brain regions, declined through childhood, and stabilized in adulthood (Figure 3d). In contrast, *CNNM2* exhibited a sustained increase in expression from infancy, reaching a plateau in late childhood and remaining stable (Figure 3e).

### Virtual drug screen

**Figure 4.**
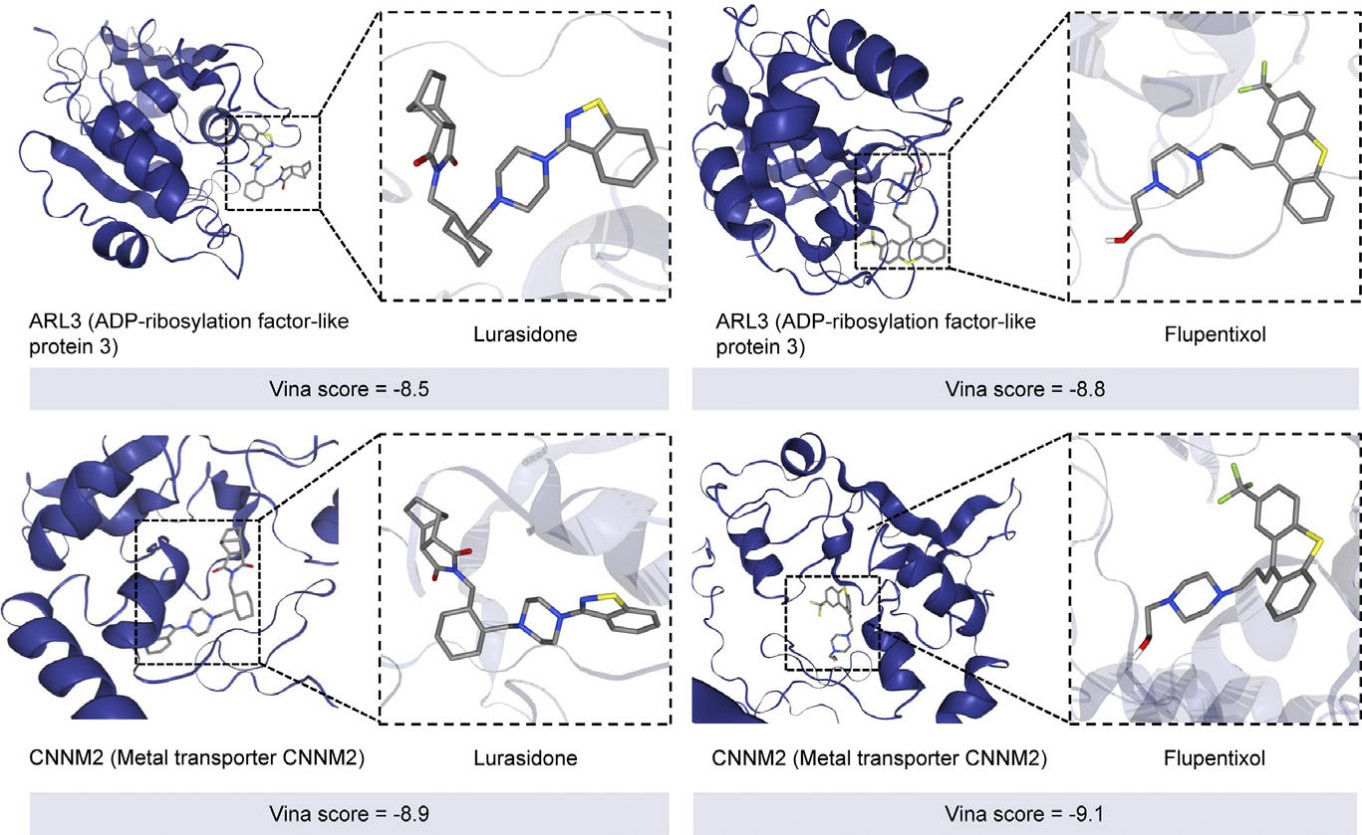
Virtual drug screen based on shared genes. Examples of virtual drug screens for ARL3 and CNNM2 with Lurasidone and Flupentixol. The corresponding Vina scores are shown.

We performed molecular docking experiments using DrugRep (http://cao.labshare.cn:10180/DrugRep/php/index.php), docking drugs with the target proteins corresponding to significant candidate proteins derived from shared genes. A total of two SNPs were found to be shared among BD, SCZ, and BD&SCZ-shared GMV deficits. These SNPs were mapped to two proteins available in DrugRep: *ARL3* and *CNNM2*. Overall, 168 distinct drugs exhibited significant binding interactions with either *ARL3* or *CNNM2* (Supplementary Table S11). Among these, rimegepant showed exceptional binding affinity for *CNNM2*, with a Vina score of −10. Similarly, Lumacaftor displayed high affinity for *ARL3*, achieving a Vina score of −10.2. Notably, 32 drugs demonstrated dual binding capability to both *ARL3* and *CNNM2*, representing promising candidates for modulating the shared molecular mechanisms linked to BD, SCZ, and GMV reduction. Within this subset, several agents with established or emerging relevance to psychiatric and neurobiological disorders stood out. Flupentixol exhibited balanced high affinity for both targets, with Vina scores of −8.8 (*ARL3*) and − 9.1 (*CNNM2*). Lurasidone showed binding to *ARL3* (Vina score = −8.5) and *CNNM2* (Vina score = −8.9) (Figure 4).

## Discussion

This study provides a comprehensive, multi-level characterization of patterns associated with shared GMV deficits in SCZ and BD, two psychiatric disorders with substantial clinical and neuroanatomical overlap. By integrating voxel-wise meta-analysis with genome-wide association analyses, PRS-GMV associations, pleiotropic SNP identification, spatiotemporal gene expression profiling, and molecular docking, we systematically examined convergent neuroanatomical regions and genetic signals linked to both disorders. Our findings highlight correlational convergence across neuroimaging and genetic levels, suggesting that SCZ and BD may partially share genetic architectures related to transdiagnostic structural brain alterations. These results provide a framework for future studies aimed at disentangling shared versus disorder-specific pathways underlying brain structural abnormalities in major psychiatric disorders.

Meta-analysis delineates shared and distinct GMV alterations in SCZ and BD. A core finding is the shared GMV reduction across SCZ and BD in several critical brain regions, notably the bilateral STP, insula, and FIOG. Previous studies have shown that the anterior insula and dorsal anterior cingulate cortex have been identified as a common neurobiological substrate across six major psychiatric disorders, including SCZ and BD (Goodkind et al., 2015). Consistent with prior research, these structural brain changes underpin both psychotic and affective symptoms in SCZ and BD (Sorella et al., 2019). Functional neuroimaging evidence further supports this view, showing that both disorders exhibit alterations in large-scale intrinsic brain networks critical for emotion processing and cognitive control (Grecucci et al., 2023; Grecucci et al., 2025). Specifically, GMV deficits are far more extensive in SCZ, spanning temporal, frontal, parietal, and occipital lobes, as well as subcortical structures and the cerebellum. This disparity aligns with SCZ’s greater cognitive impairment and functional disability (Bortolato et al., 2015; Li et al., 2020). A recent study further indicates that SCZ displays the most pronounced GM-WM abnormalities in both extent and severity when transposed independent vector analysis is applied to gray and white matter images, with BD lying in an intermediate position (Grecucci et al., 2025). Moreover, the cerebellar GMV reduction exclusive to SCZ in our study echoes prior work highlighting cerebellar contributions to emotional and cognitive processing in severe psychopathology (Sorella et al., 2019). Besides, we note that several core regions identified in prior literature – such as the fronto-temporal and limbic regions – show substantial overlap with our findings, supporting the robustness and consistency of the shared GMV deficit patterns across independent datasets and analytical frameworks. Meta-regression revealed disease-specific dynamics: no association between GMV changes and age/illness duration in BD, whereas SCZ showed negative correlations in multiple regions, such as cerebellum, FIOG, and STP. These findings suggest a potential pattern of structural decline in SCZ that warrants further longitudinal investigation.

GWAS analysis identified 14 independent SNPs linked to SCZ and BD-shared GMV deficits. Notably, rs2274224 (exonic in *PLCE1/PLCE1-AS1*, CADD = 17.35) and rs10784447 (intronic in *MSRB3*, CADD = 20.6) exhibit high functional impact. *PLCE1* is associated with calcium signaling (Garland-Kuntz et al., 2018; Li et al., 2018), a pathway implicated in both SCZ and BD (Kabir, Martínez-Rivera, & Rajadhyaksha, 2017), while *MSRB3* regulates oxidative stress responses (Kwak et al., 2012), critical for neuronal survival. Intriguingly, rs10786670 maps to *SUFU*, a gene previously linked to SCZ; functional studies show that *SUFU* dysregulation impairs neurogenesis and dendritic spine formation, which may drive GMV loss (Wang et al., 2023). In addition, the PRS-GMV association analyses illuminate polygenetic contributions to GMV in brain regions with shared reduction in SCZ and BD. For SCZ, a modest negative correlation between SCZ-PRS and GMV aligns with prior work showing that SCZ genetic risk is associated with structural brain alterations, like reduced GMV in the frontal and temporal regions (Haijma et al., 2013; Sugihara et al., 2017). This may support SCZ’s polygenic architecture, driving neuroanatomical vulnerability in shared deficit areas.

Pleiotropic analysis identified two significant SNPs (rs11191368 in *ARL3* and rs79668541 in *CNNM2*) shared among BD, SCZ, and BD&SCZ-shared GMV deficits. *ARL3* is a member of the small GTPase family, and it localizes to cilia and microtubules and plays a role in the formation of axons and cilia. A SNP located within an intron of *ARL3* was significantly associated with SCZ in a Han Chinese population (Yu et al., 2017), and decreased expression of *ARL3* has been associated with SCZ (Dang, Liu, Zhang, & Luo, 2023) and in a proteome-wide association study (Liu, Li, & Luo, 2021). *CNNM2* mediates magnesium transport; magnesium dyshomeostasis is implicated in mood disorders and SCZ pathophysiology, with animal models showing *CNNM2* disruption alters neural excitability (Zhou et al., 2024).

The molecular docking analysis identifies lurasidone and flupentixol as potential candidate drugs. Lurasidone is a second-generation antipsychotic with high affinity for D2, 5-HT2A, and 5-HT7 receptors, moderate 5-HT1A partial agonism, and moderate binding to *CNNM2* and *ARL3*, conferring antipsychotic, mood-stabilizing, and procognitive effects (DelBello et al., 2017; Koukopoulos et al., 2025). Flupentixol, a dopamine-receptor-blocking thioxanthene, is effective against both SCZ and BD by reducing positive symptoms and manic relapses (Ahlfors et al., 1981; Bailey & Taylor, 2019; Palaniyappan & Liddle, 2025).

Several limitations should be considered when interpreting these findings. First, despite rigorous inclusion criteria, variability in imaging protocols and clinical assessments across studies may have introduced methodological heterogeneity that could influence the meta-analytic findings. Second, although formal a priori power calculations are not feasible for coordinate-based neuroimaging meta-analyses, the large aggregated sample sizes in this study provide adequate power to detect consistent GMV alterations across studies. Third, potential sampling biases, including differences in recruitment strategies and demographic composition, should be acknowledged and may contribute to residual variability in the results. Fourth, most included genetic data were derived from individuals of European ancestry, which may limit the generalizability of the findings to other populations. Fifth, associations between PRS and brain structural measures were weak in SCZ and did not reach statistical significance in BD. This likely reflects the limited sensitivity of current PRS methods to detect small genetic effects on intermediate neuroimaging phenotypes, particularly in the presence of greater phenotypic and genetic heterogeneity in BD. Finally, molecular docking only provides in-silico binding affinity, which requires further verification through clinical experiments.

In summary, this study provides a comprehensive, multi-level characterization of the neurobiological substrates underlying the shared GMV deficits observed in SCZ and BD. We delineated convergent brain regions and genetic mechanisms contributing to the transdiagnostic structural abnormalities of both disorders. These findings highlight a shared neurogenetic architecture that bridges structural brain alterations and molecular pathways, offering new insights into the common pathophysiology of SCZ and BD. Ultimately, our work lays a foundation for future transdiagnostic research and precision medicine strategies targeting overlapping mechanisms across major psychiatric disorders.

## Supporting information

10.1017/S0033291726104334.sm001 Xie et al. supplementary material Xie et al. supplementary material

## Supplementary material

The supplementary material for this article can be found at http://doi.org/10.1017/S0033291726104334.
