# Supplementary material for: Shared genetic architecture of gray matter deficits in schizophrenia and bipolar disorder: evidence from structural neuroimaging–genetic analyses
Source: Psychol Med. 2026 May 14;56:e149. doi: 10.1017/S0033291726104334 (PMC13200159; doi:10.1017/S0033291726104334)
Supplement: Xie et al. supplementary material [file S0033291726104334sup001.pdf]

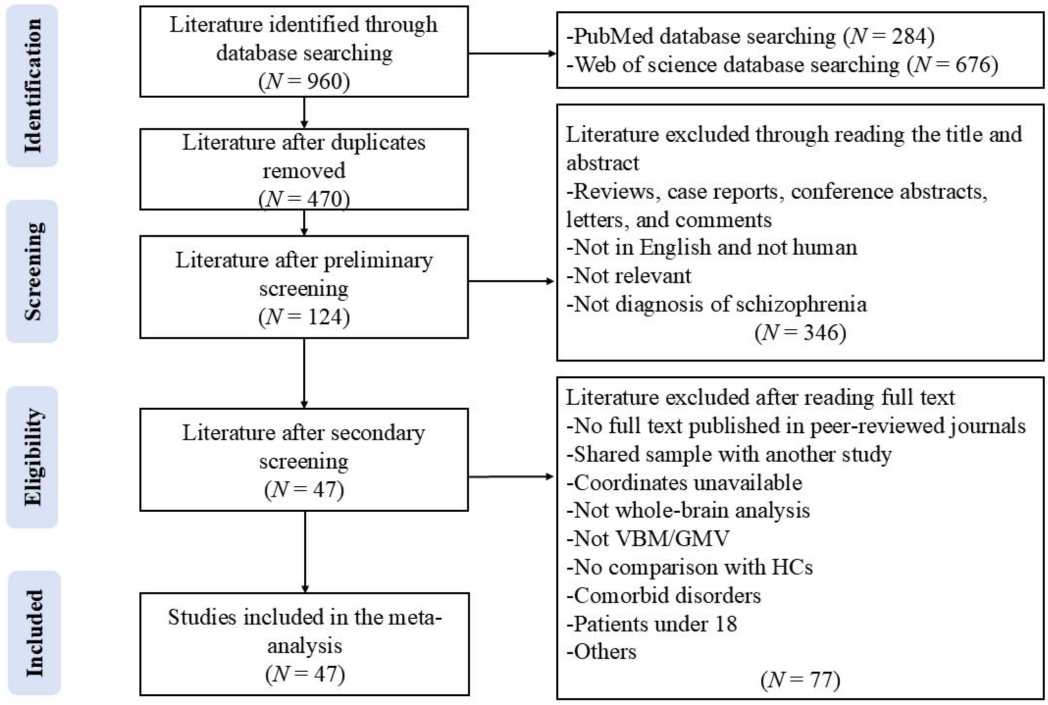

Supplementary Figure 1. The flowchart of literature search and selection in the meta-analysis of BD. Abbreviations: BD, bipolar disorder; GMV, gray matter volume; HCs, health controls; *N*, number; VBM, voxel-based morphometry.

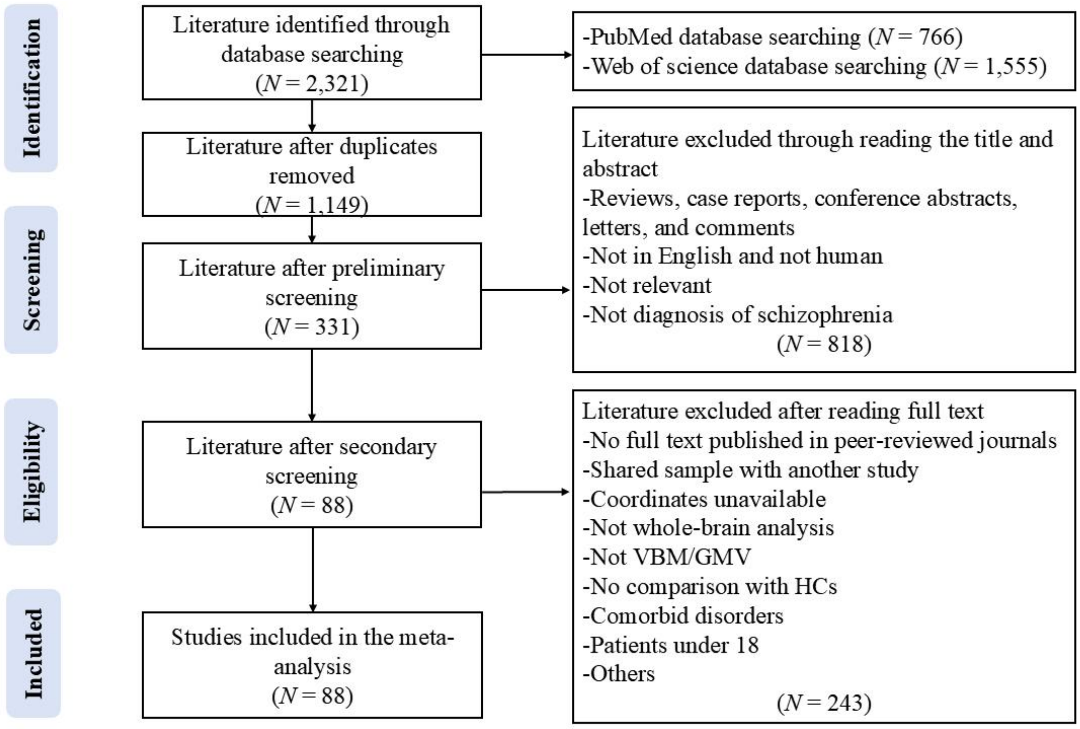

Supplementary Figure 2. The flowchart of literature search and selection in the meta-analysis of SCZ. Abbreviations: GMV, gray matter volume; HCs, health controls; *N*, number; SCZ, schizophrenia; VBM, voxel-based morphometry.

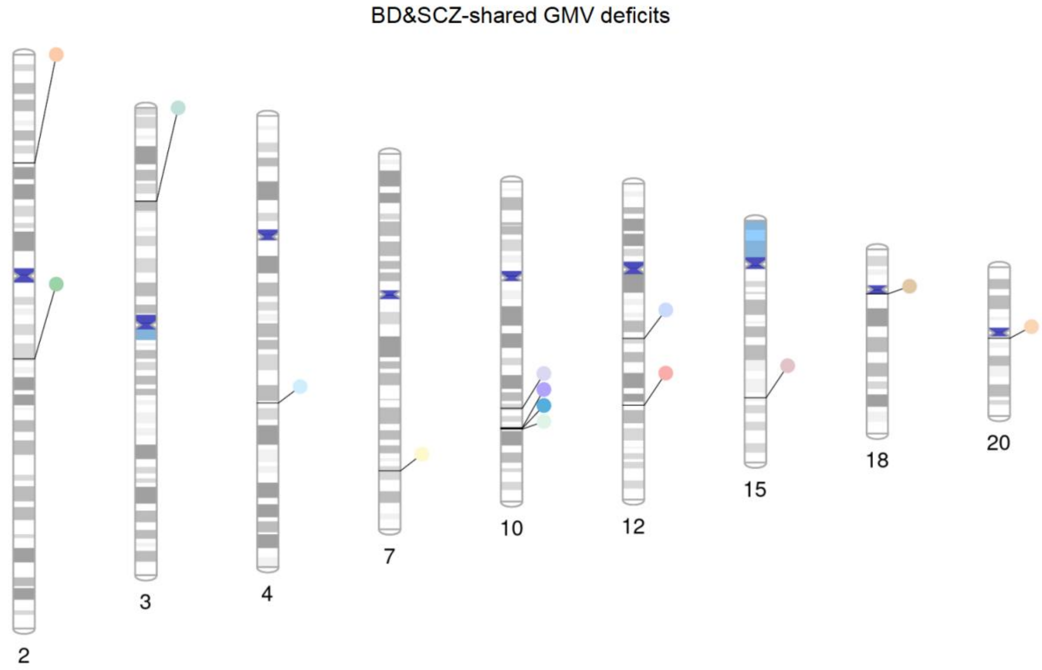

Supplementary Figure 3. The genomic location of pleiotropic SNPs associated with BD&SCZ-shared GMV deficits. This figure illustrates the genomic locations of loci ($P < 5 \times 10^{-8}$) associated with GMV deficits shared between SCZ and BD. Abbreviations: BD, bipolar disorder; GMV, grey matter volume; SCZ, schizophrenia.

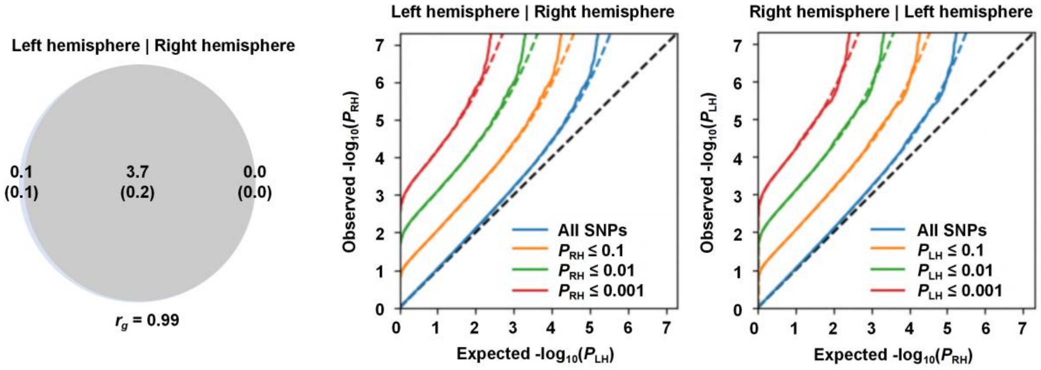

Supplementary Figure 4. Pleiotropic genetic overlap between left and right hemispheric gray matter volumes. Venn diagrams illustrate the numbers of shared (gray) causal SNPs between left and right hemispheric gray matter volumes. Conditional Q-Q plots display the expected versus observed -log$_{10}$(P) values for the left hemisphere conditioned on the significance of association with the right hemisphere, and vice versa. Abbreviations: LH, left hemisphere; RH, right hemisphere.

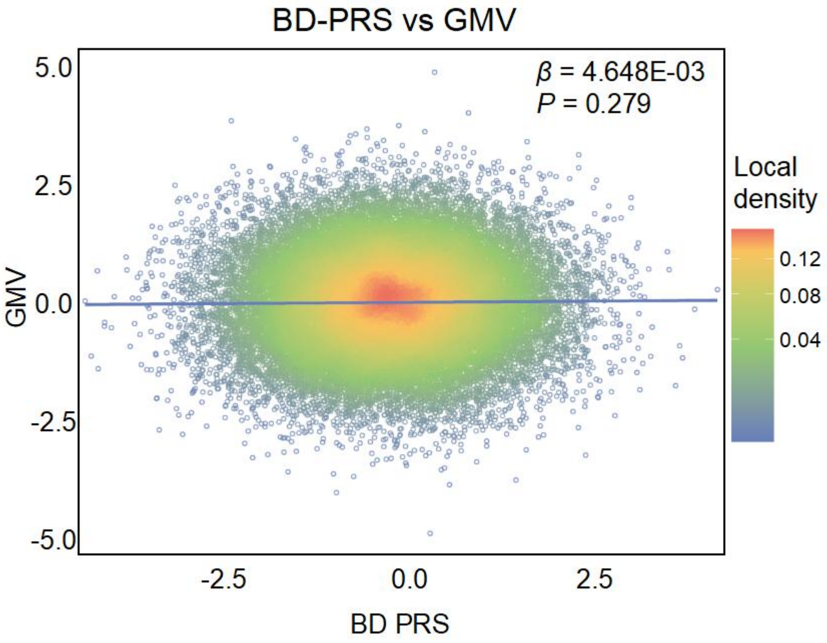

Supplementary Figure 5. Associations between BD PRS and normalized GMV. The scatter plot (with local density coloring from light blue to yellow and hollow circle markers) illustrates the correlation between BD PRS and normalized GMV. Abbreviations: BD, bipolar disorder; GMV, grey matter volume; PRS, polygenic risk scores.

Supplementary Table 1. The quality assessment checklist and the scores of the included BD studies.

	Category 1: Participants				Category 2: Methods for image acquisition and analysis				Category 3: Results and conclusions		
Score (0/0.5/1)	1. Patients were evaluated prospectively, specific diagnostic criteria were applied, and demographic data were reported.	2. Healthy comparison participants were evaluated prospectively; psychiatric and medical illnesses were excluded.	3. Important variables (e.g., age, sex, illness duration, onset, medication status, comorbidity, severity of illness) were checked either by stratification or statistically.	4. Sample size per group > 10.	5. Whole brain analysis was automated with no a priori regional selection.	6. Coordinates reported in a standard space.	7. The imaging technique used was clearly described so that it could be reproduced.	8. Measurements were clearly described so that they could be reproduced.	9. Statistical parameters for significant and important nonsignificant differences were provided.	10. Conclusions were consistent with the results obtained and the limitations were discussed.	*Total*
Adler et al. (2005)	1	1	1	1	1	1	1	1	1	0.5	9.5
Alonso-Lana et al. (2016)	1	1	1	1	1	1	1	1	1	1	10
Altamura et al.	1	1	1	1	1	1	1	1	1	1	10

(2017)											
Ambrosi et al. (2013)	1	1	1	1	1	1	1	1	1	1	10
Baez et al. (2019)	1	1	1	1	1	1	1	1	1	1	10
Brown et al. (2011)	1	1	1	1	1	1	1	1	1	1	10
Bruno et al. (2004)	1	0.5	1	1	0	1	1	1	1	1	9.5
Cai et al. (2015)	1	1	1	1	1	1	1	1	1	1	10
Chen et al. (2007)	1	1	1	1	1	1	1	1	1	1	10
Chen et al. (2018)	1	1	1	1	1	1	1	1	1	1	10
Eker et al. (2014)	1	1	1	1	1	1	1	1	1	1	10
Emsell et al. (2013)	1	1	1	1	1	1	1	1	1	0.5	9.5
Frangou et al. (2011)	1	1	1	1	1	1	0.5	0.5	1	0.5	8.5
Goikolea et al. (2019)	1	1	1	1	1	1	0.5	0.5	1	1	9
Hajek et al. (2014)	1	1	1	1	1	1	1	1	1	1	10
Ivleva et al. (2013)	1	0.5	1	1	1	1	0.5	0.5	1	1	8.5
Kandilarova et al. (2019)	1	1	1	1	1	1	1	0.5	1	1	9.5
Lee et al. (2020)	1	1	1	1	1	1	1	1	1	1	10
Li et al. (2011)	1	0	1	1	1	1	1	1	1	1	10

Lochhead et al. (2004)	1	1	1	1	1	1	1	1	1	1	10
McDonald et al. (2005)	1	1	1	1	1	1	1	1	1	1	10
Metin et al. (2023)	1	1	1	1	1	1	1	0.5	1	1	9.5
Miola et al. (2022)	1	1	1	1	1	1	0.5	0.5	1	1	9
Molina et al. (2011)	1	1	1	1	1	1	1	1	1	1	10
Narita et al. (2011)	1	1	1	1	1	1	1	1	1	1	10
Nenadic et al. (2015)	1	1	1	1	1	1	1	0.5	1	1	9.5
Nery et al. (2015)	1	1	1	1	1	1	1	1	1	1	10
Neves et al. (2015)	1	1	1	1	1	1	1	1	1	1	10
Nugent et al. (2006)	1	1	1	1	1	1	1	1	1	1	10
Ota et al. (2016)	1	1	1	1	1	1	0.5	0.5	1	1	9
Poletti et al. (2016)	1	1	1	1	1	1	1	1	1	1	10
Quide et al. (2020)	1	1	1	1	1	1	1	1	1	1	10
Rocha-Rego et al. (2014)	1	1	1	1	1	1	1	0.5	1	0.5	9

Sani et al. (2016)	1	1	1	1	1	1	1	1	1	1	10
Sarıçiçek et al. (2015)	1	1	1	1	1	1	1	1	1	1	10
Scherk et al. (2008)	1	1	1	1	1	1	1	1	1	0.5	9.5
Song et al. (2015)	1	1	1	1	1	1	1	1	1	1	10
Song et al. (2020)	1	1	1	1	1	1	1	1	1	1	10
Sun et al. (2020)	1	1	1	1	1	1	1	1	1	1	10
Tang et al. (2014)	1	1	1	1	1	1	1	1	1	1	10
Thomas-Odenthal et al. (2024)	1	1	1	1	1	1	1	1	1	1	10
Thiel et al. (2024)	1	1	1	1	1	1	0.5	0.5	1	1	9
Watson et al. (2012)	1	1	1	1	1	1	1	1	1	1	10
Yang et al. (2022)	1	1	1	1	1	1	1	0.5	1	1	9.5
Yi*p* et al. (2013)	1	1	1	1	1	1	1	1	1	1	10
Yüksel et al. (2012)	1	1	1	1	1	1	1	1	1	1	10
Zhang et al. (2021)	1	1	1	1	1	1	1	1	1	1	10

Supplementary Table 2. The quality assessment checklist and the scores of the included SCZ studies.

	Category 1: Participants				Category 2: Methods for image acquisition and analysis				Category 3: Results and conclusions		
Score (0/0.5/1)	1. Patients were evaluated prospectively, specific diagnostic criteria were applied, and demographic data were reported.	2. Healthy comparison participants were evaluated prospectively, psychiatric and medical illnesses were excluded.	3. Important variables (e.g., age, sex, illness duration, onset, medication status, comorbidity, severity of illness) were checked either by stratification or statistically.	4. Sample size per group > 10.	5. Whole brain analysis was automated with no a priori regional selection.	6. Coordinates reported in a standard space.	7. The imaging technique used was clearly described so that it could be reproduced.	8. Measurements were clearly described so that they could be reproduced.	9. Statistical parameters for significant and important nonsignificant differences were provided.	10. Conclusions were consistent with the results obtained and the limitations were discussed.	Total
Adamu et al. (2023)	1	1	1	1	1	1	1	1	1	1	10
Ananth et al. (2002)	1	1	1	1	1	1	1	1	1	0.5	9.5

Anderson et al. (2015)	1	1	1	1	1	1	1	1	1	1	10
Antonova et al. (2005)	1	1	1	1	1	1	1	1	1	1	10
Asami et al. (2012)	1	1	1	1	1	1	1	1	1	1	10
Assche et al. (2024)	1	1	1	1	1	1	1	1	1	1	10
Bagary et al. (2003)	1	1	1	1	1	1	1	1	1	1	10
Berge et al. (2010)	1	0	1	1	1	1	1	1	1	1	9
Bonidi et al. (2025)	1	1	1	1	1	1	1	1	1	1	10
Bonilha et al. (2008)	1	1	1	1	1	1	1	1	1	0.5	9.5
Borgwardt et al. (2010)	1	0.5	1	1	1	1	1	1	1	1	9.5
Bose et al. (2009)	1	0	1	1	1	1	1	1	1	0.5	8.5
Brown et al. (2011)	1	0	1	1	1	1	1	1	1	1	9
Cascella et al. (2010)	1	1	1	1	1	1	1	1	1	1	10

Chow et al. (2011)	1	0	1	1	1	1	1	1	1	1	9
Cooke et al. (2008)	1	1	1	1	1	1	1	1	1	0.5	9.5
Ellison-Wright et al. (2014)	1	1	1	1	1	1	1	1	1	1	10
Euler et al. (2009)	1	1	1	1	1	1	1	1	1	1	10
Ferri et al. (2012)	1	1	1	1	1	1	1	1	1	0.5	9.5
Filippi et al. (2014)	1	1	1	1	1	1	1	1	1	1	10
Frascarelli et al. (2015)	1	0	1	1	1	1	1	1	1	1	9
Fukuta et al. (2013)	1	1	1	1	1	1	1	1	1	1	10
Garcia et al. (2024)	1	1	1	1	1	1	1	1	1	1	10
Guo et al. (2013)	1	1	1	1	1	1	1	1	1	1	10
Gou et al. (2022)	1	1	1	1	1	1	1	1	1	1	10
Honea et al. (2008)	1	1	1	1	1	1	1	1	1	1	10
Hooker et al. (2011)	1	0	1	1	1	1	1	1	1	1	9

Horacek et al. (2012)	1	1	1	1	1	1	1	1	1	1	10
Horn et al. (2010)	1	1	1	1	1	1	1	1	1	0.5	9.5
Huang et al. (2015)	1	0	1	1	1	1	1	1	1	1	9
Huang et al. (2017)	1	1	1	1	1	1	1	1	1	1	10
Hýža et al. (2014)	1	1	1	1	1	1	1	1	1	1	10
Ivleva et al. (2013)	1	1	1	1	1	1	1	1	1	1	10
Jayakumar et al. (2005)	1	1	1	1	1	1	1	1	1	0.5	9.5
Kašpárek et al. (2007)	1	1	1	1	1	1	1	1	1	1	10
Katz et al. (2016)	1	0	1	1	1	1	1	1	1	1	9
Kim et al. (2017)	1	0	1	1	1	1	1	1	1	1	9
Koelkebeck et al. (2019)	1	1	1	1	1	1	1	1	1	1	10
Kong et al. (2015)	1	1	1	1	1	1	1	1	1	1	10

Lee et al. (2020)	1	1	1	1	1	1	1	1	1	1	10
Li et al. (2020)	1	1	1	1	1	1	1	1	1	1	10
Liao et al. (2015)	1	1	1	1	1	1	1	1	1	1	10
Lu et al. (2024)	1	1	1	1	1	1	1	1	1	1	10
Ma et al. (2021)	1	1	1	1	1	1	1	1	1	1	10
Maggioni et al. (2017)	1	0	1	1	1	1	1	1	1	1	9
McDonald et al. (2005)	1	1	1	1	1	1	1	1	1	1	10
Molina et al. (2011)	1	1	1	1	1	1	1	1	1	1	10
Nakamura et al. (2013)	1	1	1	1	1	1	1	1	1	1	10
Nemoto et al. (2020)	1	1	1	1	1	1	1	1	1	1	10
Neugebauer et al. (2019)	1	1	1	1	1	1	1	1	1	1	10
Oertel-Knöchel et al. (2012)	1	1	1	1	1	1	1	1	1	0.5	9.5

Onay et al. (2017)	1	1	1	1	1	1	1	1	1	1	10
Ota et al. (2017)	1	1	1	1	1	1	1	1	1	1	10
Palaniyappan et al. (2012)	1	1	1	1	1	1	1	1	1	0.5	9.5
Picado et al. (2015)	1	1	1	1	1	1	1	1	1	1	10
Quidé et al. (2020)	1	1	1	1	1	1	1	1	1	1	10
Ren et al. (2013)	1	1	1	1	1	1	1	1	1	0.5	9.5
Rigucci et al. (2013)	1	1	1	1	1	1	1	1	1	1	10
Rootes-Murdy et al. (2021)	1	1	1	1	1	1	1	1	1	1	10
Rose et al. (2014)	1	0.5	1	1	1	1	1	1	1	0.5	9
RužićBaršić et al. (2021)	1	1	1	1	1	1	1	1	1	0.5	9.5
Salgado-Pineda et al. (2004)	1	1	1	1	1	1	1	1	1	1	10
Salgado-Pineda et al. (2011)	1	1	1	1	1	1	1	1	1	1	10

Sanjuán et al. (2021)	1	0	1	1	1	1	1	1	1	1	9
Sarró t al. (2013)	1	1	1	1	1	1	1	1	1	0.5	9.5
Schiffer et al. (2013)	1	0.5	1	1	1	1	1	1	1	1	9.5
Schuster et al. (2012)	1	1	1	1	1	1	1	1	1	1	10
Siddi et al. (2019)	1	1	1	1	1	1	1	1	1	1	10
Singh et al. (2018)	1	1	1	1	1	1	1	1	1	1	10
Song et al. (2015)	1	1	1	1	1	1	1	1	1	1	10
Stegmayer et al. (2016)	1	1	1	1	1	1	1	1	1	1	10
Tan et al. (2015)	1	1	1	1	1	1	1	1	1	1	10
Tikàsz et al. (2019)	1	1	1	1	1	1	1	1	1	1	10
Tomelleri et al. (2009)	1	1	1	1	1	1	1	1	1	0.5	9.5
Torres et al. (2016)	1	1	1	1	1	1	1	1	1	1	10
Tregellas et al. (2007)	1	1	1	1	1	1	1	1	1	0.5	9.5

Tseng et al. (2021)	1	1	1	1	1	1	1	1	1	1	10
van Tol et al. (2014)	1	0.5	1	1	1	1	1	1	1	1	9.5
Walther et al. (2024)	1	1	1	1	1	1	1	1	1	1	10
Watson et al. (2012)	1	1	1	1	1	1	1	1	1	1	10
Wu et al. (2018)	1	1	1	1	1	1	1	1	1	0.5	9.5
Wu et al. (2022)	1	1	1	1	1	1	1	1	1	1	10
Xie et al. (2021)	1	1	1	1	1	1	1	1	1	1	10
Yang et al. (2019)	1	1	1	1	1	1	1	1	1	1	10
Yang et al. (2022)	1	1	1	1	1	1	1	1	1	1	10
Yang et al. (2023)	1	1	1	1	1	1	1	1	1	0.5	9.5
Yüksel et al. (2012)	1	1	1	1	1	1	1	1	1	1	10
Zhang et al. (2017)	1	1	1	1	1	1	1	1	1	1	10

Supplementary Table 3. Demographic and clinical characteristics of the studies included in BD.

Study	BD			HC			Duration (y)
	Sample size	Male	Mean age (y)	Sample size	Male	Mean age (y)	
Adler et al. (2005)[1]	32	19	31.2	27	12	30.5	8.7
Alonso-Lana et al. (2016)[2]	33	18	44.1	28	12	44	16.8
Altamura et al. (2017)[3]	17	4	38.7	27	16	34	11.4
Ambrosi et al. (2013)[4]	20	5	42	21	6	34.6	12.6
Baez et al. (2019)[5]	13	3	61.9	22	7	62.5	>10
Brown et al. (2011)[6]	15	7	46.2	21	10	45	18.9
Bruno et al. (2004)[7]	39	13	38.8	35	10	34.8	13.2
Cai et al. (2015)[8]	23	16	25.7	23	13	28.2	6.1
Chen et al. (2007)[9]	24	6	38.2	25	7	38.4	14.2
Chen et al. (2018)[10]	43	17	27.9	47	22	29.7	2.9
Eker et al. (2014)[11]	28	16	36.4	30	10	34.7	16.3
Emsell et al. (2013)[12]	60	31	42	60	31	42	13
Frangou et al. (2011)[13]	47	21	46.2	71	36	39.8	20
Goikolea et al. (2019)[14]	31	16	30.5	31	16	31.1	3.3
Hajek et al. (2014)[15]	33	16	51.6	11	4	43.1	27.9
Ivleva et al. (2013)[16]	115	36	35.4	200	92	39.8	15.2
Kandilarova et al. (2019)[17]	11	3	43.6	50	13	42.6	15.5
Lee et al. (2020)[18]	65	29	35.1	65	28	34.5	13.5
Li et al. (2011)[19]	24	15	28.4	36	21	26.6	6
Lochhead et al. (2004)[20]	11	6	38.2	31	16	36	13.9
McDonald et al. (2005)[21]	37	15	40.7	52	24	39.3	17.8
Metin et al. (2023)[22]	37	24	44.9	27	17	40.5	/

Miola et al. (2022a)[23]	24	18	43.2	45	25	41.5	17.8
Miola et al. (2022b)[23]	30	19	39.5	45	25	41.5	12.8
Molina et al. (2011)[24]	19	12	38.3	24	16	34.6	12
Narita et al. (2011a)[25]	14	8	40.2	84	48	41.1	8.6
Narita et al. (2011b)[25]	17	9	41.4	84	48	41.1	6.2
Nenadic et al. (2015)[26]	17	9	37.7	34	18	34.3	9.9
Nery et al. (2015)[27]	25	8	35.7	27	11	31.2	13.6
Neves et al. (2015)[28]	21	10	39	21	10	37.9	11.6
Nugent et al. (2006a)[29]	20	5	41	65	19	38	23
Nugent et al. (2006b)[29]	16	5	37	65	19	38	17
Ota et al. (2016)[30]	43	22	38.6	229	61	45.6	11.7
*P*oletti et al. (2016)[31]	206	72	46.2	136	68	33.3	15.5
Quide et al. (2020)[32]	65	19	35.9	61	34	36	13.8
Rocha-Rego et al. (2014)[33]	26	12	41.5	26	12	41.3	15.8
Sani et al. (2016)[34]	78	38	44.6	78	38	44.4	17.3
Sarıçiçek et al. (2015)[35]	28	18	36.3	29	13	33.6	10.6
Scherk et al. (2008)[36]	35	18	43.3	32	12	33.7	14.4
Song et al. (2015)[37]	44	19	34.8	35	11	33.9	7.4
Sun et al. (2020)[38]	30	15	36.3	31	20	33.6	/
Tang et al. (2014)[39]	27	10	32	27	11	32.6	4.2
Thomas-Odenthal et al. (2024)[40]	87	43	27.6	115	48	27	9.7
Thiel et al. (2024a)[41]	73	31	41.8	136	59	42.5	/
Thiel et al. (2024b)[42]	63	30	40.5	136	59	42.5	/
Watson et al. (2012)[43]	24	8	36	24	8	35.6	/
Yang et al. (2022)[44]	42	23	32.9	95	47	30.2	6.4
Yi*p* et al. (2013)[45]	38	20	20.9	37	20	21.2	/

Yüksel et al. (2012)[46]	28	17	32.9	43	28	36.4	/
Zhang et al. (2021)[47]	13	6	31	20	10	31.7	9

Supplementary Table 4. Demographic and clinical characteristics of the studies included in SCZ.

Study	SCZ			HC			Duration (y)
	Sample size	Male	Mean age (y)	Sample size	Male	Mean age (y)	
Adamu et al. (2023)[48]	72	58	38.2	74	51	35.8	16
Ananth et al. (2002)[49]	20	10	37.8	20	10	38.6	15.9
Anderson et al. (2015a)[50]	18	14	32.2	20	17	33.3	10
Anderson et al. (2015b)[50]	19	14	33.3	20	17	33.3	13
Anderson et al. (2015c)[50]	15	13	34.3	20	17	33.3	11.4
Antonova et al. (2005)[51]	45	27	40.5	43	25	33.7	16.9
Asami et al. (2012)[52]	33	28	22.5	36	30	22.9	0.4
Bagary et al. (2003)[53]	30	19	27.3	30	18	28.9	0.4
Berge et al. (2010)[54]	21	12	24.8	20	8	25.3	/
Bonidi et al. (2025)[55]	74	60	37.4	91	65	38.5	14.8
Bonilha et al. (2008)[56]	14	11	40	13	11	35	/
Borgwardt et al. (2010)[57]	28	22	37.7	34	24	39.3	16.8
Bose et al. (2009)[58]	34	34	39.5	33	33	39.5	12
Brown et al. (2011)[6]	17	8	44.8	21	10	45	19.1
Cascella et al. (2010)[59]	50	37	40.9	90	43	46.3	16.7
Chow et al. (2011)[60]	29	11	30.7	34	17	27.8	8.3
Cooke et al. (2008)[61]	52	40	38.4	30	24	32.1	13.9
Ellison-Wright et al. (2014)[62]	16	/	/	19	/	/	/
Euler et al. (2009)[63]	19	14	27.2	20	/	/	/
Ferri et al. (2012)[64]	19	14	27.2	19	11	28.7	0.6
Filippi et al. (2014)[65]	43	24	29.3	17	6	30.7	0.7
Frascarelli et al. (2015a)[66]	18	8	29.2	24	9	30.3	4.9

Frascarelli et al. (2015b)[66]	15	9	42.7	24	9	30.3	17.2
Fukuta et al. (2013)[67]	40	0	45.6	50	0	45	23
Garcia et al. (2024)[68]	128	94	26.5	78	52	26.4	NA
Guo et al. (2013a)[69]	27	16	25.1	30	14	25.6	0.1
Guo et al. (2013b)[69]	30	16	25.7	30	14	25.6	1.2
Gou et al. (2022a)[70]	31	31	30.6	43	43	31.8	6.1
Gou et al. (2022b)[70]	39	39	29.2	43	43	31.8	4.5
Honea et al. (2008)[71]	169	132	36.4	212	103	33.3	/
Hooker et al. (2011)[72]	21	17	44.3	17	13	43.8	24.5
Horacek et al. (2012)[73]	44	22	30.8	56	23	27.9	6.6
Horn et al. (2010)[74]	20	13	30.1	20	13	30.1	/
Huang et al. (2015a)[75]	18	10	22.6	18	9	25.1	0.5
Huang et al. (2015b)[75]	18	9	22.7	18	9	25.1	1
Huang et al. (2017a)[76]	18	12	22.5	26	17	23.2	0.7
Huang et al. (2017b)[76]	24	14	24.3	26	17	23.2	0.8
Hýža et al. (2014)[77]	24	11	32.8	24	11	31.8	9.2
Ivleva et al. (2013a)[16]	146	102	35.8	200	92	39.8	14.3
Ivleva et al. (2013b)[16]	90	37	36.3	200	92	39.8	16.5
Jayakumar et al. (2005)[78]	18	9	24.9	18	9	25.7	0.9
Kašpárek et al. (2007)[79]	22	22	23.7	18	18	24.1	0.8
Katz et al. (2016)[80]	24	24	31.2	32	32	29.8	/
Kim et al. (2017)[81]	22	12	31.7	22	12	31.6	9.2
Koelkebeck et al. (2019)[82]	163	89	33.4	203	122	31.3	9.3
Kong et al. (2015)[83]	22	16	54	20	12	52.8	31.5
Lee et al. (2020)[18]	65	36	37	65	28	34.5	15.5
Li et al. (2020)[84]	86	46	23.5	86	45	24	1.2

Liao et al. (2015)[85]	93	57	27	99	53	25.8	4.5
Lu et al. (2024a)[86]	45	45	30.8	53	53	31.2	6.3
Lu et al. (2024b)[86]	45	45	27.9	53	53	31.2	4.8
Ma et al. (2021)[87]	64	38	26.7	65	33	25.3	/
Maggioni et al. (2017)[88]	243	152	33.2	383	195	30.4	7.1
McDonald et al. (2005)[21]	25	18	37.3	52	24	39.3	17.4
Molina et al. (2011)[24]	38	26	34.4	24	16	34.6	9.8
Nakamura et al. (2013)[89]	34	20	24.7	51	30	23.9	/
Nemoto et al. (2020)[90]	95	57	29.8	95	57	29.9	8.4
Neugebauer et al. (2019)[91]	18	11	36.9	19	12	35.8	12.6
Oertel-Knöchel et al. (2012)[92]	31	16	38	37	17	39.4	13.7
Onay et al. (2017)[93]	20	10	36.5	16	7	34.4	10.7
Ota et al. (2017)[94]	37	17	36.2	62	17	40.6	13.5
Palaniyappan et al. (2012)[95]	57	50	26.1	41	39	28	/
Picado et al. (2015)[96]	20	11	35.9	20	12	33.2	/
Quidé et al. (2020)[32]	60	36	41.2	61	34	36	18.3
Ren et al. (2013)[97]	100	41	24.3	100	41	24.4	0.5
Rigucci et al. (2013)[98]	19	12	22.2	18	11	23.4	0.7
Rootes-Murdy et al. (2021)[99]	65	53	38.1	79	62	37.7	/
Rose et al. (2014)[100]	163	55	38.5	150	84	33.5	15.2
RužićBaršić et al. (2021)[101]	57	26	44.6	50	25	35.8	13.8
Salgado-Pineda et al. (2004)[102]	14	7	25.1	14	7	25.1	1.7
Salgado-Pineda et al. (2011)[103]	14	9	34.6	14	9	37.3	14
Sanjuán et al. (2021)[104]	61	61	31.9	18	18	38.2	/
Sarró t al. (2013)[105]	81	59	42.9	61	44	40.7	21.4
Schiffer et al. (2013)[106]	23	23	35.7	25	25	33	12.6

Schuster et al. (2012)[107]	27	14	59.9	40	17	62.2	29.2
Siddi et al. (2019)[108]	24	14	41.1	26	15	36.9	13.2
Singh et al. (2018)[109]	28	12	33.9	28	14	31.4	9.2
Song et al. (2015)[37]	71	29	35.6	35	11	33.9	7.1
Stegmayer et al. (2016)[110]	45	28	38.2	44	26	38.8	/
Tan et al. (2015)[111]	18	11	40.5	17	10	41.2	15.9
Tikàsz et al. (2019)[112]	47	47	34.4	23	23	31.9	12.2
Tomelleri et al. (2009)[113]	70	45	39.7	79	41	40.3	14.1
Torres et al. (2016)[114]	161	111	30.4	151	87	30.6	5
Tregellas et al. (2007)[115]	32	21	39.8	32	14	35.3	/
Tseng et al. (2021)[116]	37	21	35.7	26	11	35.6	12.2
Van Assche et al. (2024)[117]	36	9	75.7	35	9	76.7	/
van Tol et al. (2014)[118]	51	44	34	51	37	36.1	8.8
Walther et al. (2022)[119]	59	29	36	42	21	37	/
Watson et al. (2012)[43]	25	19	28.8	25	19	28.2	/
Wu et al. (2018)[120]	34	20	31.3	29	13	29.4	6.9
Wu et al. (2022)[121]	143	63	32.8	107	46	32.4	7.4
Xie et al. (2021)[122]	30	13	30.3	33	13	32	1.8
Yang et al. (2019)[123]	37	21	42	28	16	40.5	18.4
Yang et al. (2022)[44]	70	31	28.4	95	47	30.2	3.6
Yang et al. (2023)[124]	25	15	23.4	25	13	22.3	/
Yüksel et al. (2012)[46]	58	38	38.7	43	28	36.4	/
Zhang et al. (2017)[125]	49	20	32.9	57	28	36.4	0.4

Supplementary Table 5. Results of meta-analysis in patients with BD and SCZ compared to HCs.

Brain region	MNI coordinate			*Z*	*P* value	Cluster size (voxels)
	x	y	z			
**BD > HCs**						
none						
**BD < HCs**						
Right STG/ Insula/ STP/ MTG/ Rolandic Operculum/ Amygdala/ FIOG	48	4	-4	-4.536	~0	2697
Left STP/ Insula/ FIOG	-46	14	-12	-5.317	~0	1206
**SCZ > HCs**						
none						
**SCZ < HCs**						
Bilateral temporal gyrus/ Frontal gyrus/ IPG/ Occipital gyrus/ Hippocampus/ Amygdala/ Fusiform/Cingulate /Insula/ Putamen/ Caudate/ Left cerebellum	48	0	-6	-15.006	~0	98742
Right cerebellum	32	-64	-50	-5.316	~0	1175

P < 0.05; Cluster >100. Abbreviations: BD, bipolar disorder; FIOG, frontal inferior operculum gyrus; IPG, Inferior parietal gyrus; MNI, Montreal Neurological Institute; MTG, Middle temporal gyrus; SCZ, schizophrenia; STG, superior temporal gyrus; STP, superior temporal pole.

Supplementary Table 6. Results of conjunction analysis in patients with BD and SCZ compared to HCs.

Brain region	MNI coordinate			*Z*	*P* value	Cluster size (voxels)
	x	y	z			
Right superior temporal gyrus/ Insula/ STP/ MTG/ Rolandic Operculum/ Amygdala/ FIOG	48	4	-4	-4.536	~0	2680
Left STP/ Insula/ FIOG	-46	14	-12	-5.317	~0	1206

$P < 0.05$; Cluster ⩾100. Abbreviations: BD, bipolar disorder; FIOG, frontal inferior orbital Gyrus; MNI, Montreal Neurological Institute; MTG, middle temporal gyrus; SCZ, schizophrenia; STG, superior temporal gyrus; STP, superior temporal pole.

Supplementary Table 7. Summary of previous studies on GMV alterations in SCZ and BD.

Study	Method	Number of SCZ	Number of BD	Disorder GMV reduction	Shared GMV alterations	Statistical threshold
Maggioni et al., 2017	VBM meta-analysis	243	176	**BD:** anterior and mesial portions of the cingulate cortex, superior temporal cortex, temporal pole, calcarine cortex, cuneus, lingual gyrus<br>**SCZ:** widespread frontal, temporal, parietal, and occipital cortices, insula, thalamus, hippocampus, parahippocampal gyrus, amygdala, portions of the cerebellum	Superior temporal cortex, anterior, middle cingulate cortex, calcarine cortex (R)	Cluster-level FWE corrected $P < 0.05$
Sun et al., 2020	VBM analysis	46	35	**BD**: frontal gyrus, insula, rectus gyrus (Bilateral); superior temporal gyrus, amygdala (R); anterior cingulate cortex (L)<br>**SCZ**: frontal gyrus, rectus gyrus (Bilateral); superior temporal gyrus, insula, amygdala, supramarginal gyrus (R); superior temporal gyrus, anterior cingulate cortex (L)	Insula, frontal regions (Bilateral); Superior temporal gyrus, amygdala (R), anterior cingulate cortex (L)	GRF-corrected (voxel $P < 0.001$, cluster $P < 0.05$); Bonferroni post hoc
Yang et al., 2022	VBM analysis	70	42	**BD**: medial orbitofrontal cortex, inferior temporal and fusiform regions, insular cortex, hippocampus, and cerebellum<br>**SCZ**: frontal lobe, temporal lobe, occipital lobe, thalamus, hippocampus, and cerebellum	Shared regions across SCZ, BD, and MDD were reported in the orbitofrontal cortex region.	Multiple comparison correction with voxel-wise $P < 0.005$ and minimum cluster extent of 298 voxels

Ulugut et al., 2022	GingerALE meta-analysis	7094	3127	**BD:** frontal lobes, anterior cingulate cortex, insula, temporal lobes, amygdala, hippocampus (Bilateral)<br>**SCZ:** cingulate cortex, frontal and temporal lobes, insula, parietal cortex, thalamus, amygdala (Bilateral); hippocampus and uncus (L)	Consistent GM alterations across all diagnostic groups highlighted included the amygdala, insula, cingulate cortex, and medial prefrontal cortex.	Cluster-level FWE corrected $P < 0.05$
Segal et al., 2023	Normative modeling	383	228	**BD**: fewer regions survived FDR correction compared to SCZ<br>**SCZ**: widespread cortical deviations	Largely a subset of SCZ-related abnormalities	Group-based permutation tests with uncorrected and FDR-corrected $P < 0.05$
Fortea et al., 2025	SDM-PSI meta-analysis	5465	3350	**BD:** prefrontal cortex, orbitofrontal cortex, temporal lobes, insula, cerebellum, striatum<br>**SCZ:** widespread cortical and subcortical regions	Heschl's gyrus, inferior frontal gyrus (R); supplementary motor area, insula, gyrus rectus (L)	FWE correction, $P < 0.05$, k ⩾ 100

Abbreviations: ALE, activation likelihood estimation; BD, bipolar disorder; FDR, false discovery rate; FWE, family-wise error; GMV, gray matter volume; MDD, major depressive disorder; SCZ, schizophrenia; SDM-PSI, seed-based *d* mapping with permutation of subject images; TFCE, threshold-free cluster enhancement; VBM, voxel-based morphometry.

Supplementary Table 8. Results of Meta-regression patients with BD and SCZ.

Variable	Brain region	MNI coordinate			*Z*	*P* value	Cluster size
		x	y	z			(voxels)
			BD				
**Mean age**	none						
**Illness duration**	none						
			SCZ				
**Mean age**	Right cerebellum	24	-62	-56	-3.166	<0.001	475
	Right FIOG	50	12	30	-3.081	0.001	338
	Left IFG	-50	30	6	-2.912	0.002	185
	Left cerebellum	-14	-70	-24	-2.269	0.012	163
	Right SMG	62	-40	36	-2.333	0.01	102
**Illness duration**	Left cerebellum	-16	-72	-24	-3.333	<0.001	904
	Left MOG	-42	-78	6	-4.879	~0	600
	Right FIOG	50	12	32	-5.487	~0	489
	Right MOG	36	-80	38	-3.675	<0.001	501
	Right STP	42	22	-28	-3.834	<0.001	491
	Bilateral SMFG	4	56	20	-3.347	<0.001	417
	Right cerebellum	30	-62	-46	-2.598	0.005	305
	Right precuneus	6	-70	36	-2.904	0.002	240
	Right Cerebellum Crus1	30	-72	-22	-2.984	0.001	207
	Right SMG	66	-38	32	-3.232	0.001	192
	Left STP	-22	6	-26	-2.526	0.006	210
	Right MFG	36	36	26	-2.78	0.003	105

P < 0.05; Cluster >100. Abbreviations: BD, bipolar disorder; FIOG, frontal inferior operculum gyrus; IFG, inferior frontal gyrus; MFG middle frontal gyrus; MNI, Montreal Neurological Institute; MOG, middle occipital gyrus; SCZ, schizophrenia; SMFG, superior medial frontal gyrus; SMG, supramarginal gyrus; STP, superior temporal pole.

Supplementary Table 9. SNPs for GMV deficits shared between BD and SCZ.

SNP	CHR	BP	*P*	Func	Nearest gene	CADD	RDB	minChrState
rs35201757	2	45846174	3.29E-09	intergenic	SRBD1	1.716	6	5
rs12614331	2	129037905	3.29E-08	intronic	HS6ST1	2.767	5	2
rs1708104	3	39534742	7.55E-10	intronic	MOBP	0.201	NA	5
rs36003774	4	121634100	6.25E-12	intronic	PRDM5	2.272	7	5
rs4269477	7	134389334	1.98E-08	intergenic	AC009276.4	4.688	5	5
rs2274224	10	96039597	1.85E-09	exonic	PLCE1:PLCE1-AS1	17.35	6	4
rs10786670	10	104270781	5.37E-10	intronic	SUFU	0.778	7	4
rs11191453	10	104659852	6.88E-09	intronic	C10orf32-ASMT:AS3MT	4.626	6	4
rs74233809	10	104913940	4.30E-09	intronic	NT5C2	4.587	4	1
rs10784447	12	65766498	8.10E-09	intronic	MSRB3	20.6	5	4
rs10859535	12	93979892	4.39E-09	intergenic	SOCS2	1.529	6	5
rs34862454	15	75101530	2.38E-09	intergenic	LMAN1L	11.23	4	2
rs118035855	18	18994683	3.96E-08	ncRNA_intronic	GREB1L:RP11-296E23.1	1.776	7	4
rs6141934	20	30017946	4.62E-08	upstream	DEFB122	1.017	5	9

Supplementary Table 10. Pleiotropic SNPs shared among BD, SCZ, and BD&SCZ-shared GMV deficits.

SNP	CHR	BP	Func	CADD	RDB	minChrState
rs11191368	10	104449444	intronic	5.017	7	4
rs79668541	10	104793904	intronic	0.274	6	5

Supplementary Table 11. Virtual drug screen based on shared genes.

Gene	Drug ID	Drug Name	Vina Score
ARL3	DB09280	Lumacaftor	-10.2
ARL3	DB00872	Conivaptan	-10
ARL3	DB06210	Eltrombopag	-10
ARL3	DB08907	Canagliflozin	-9.8
ARL3	DB11652	Tucatinib	-9.5
ARL3	DB04861	Nebivolol	-9.5
ARL3	DB01100	Pimozide	-9.4
ARL3	DB11799	Bictegravir	-9.3
ARL3	DB12457	Rimegepant	-9.3
ARL3	DB04868	Nilotinib	-9.3
ARL3	DB00941	Hexafluronium	-9.3
ARL3	DB12877	Oxatomide	-9.3
ARL3	DB13520	Metergoline	-9.2
ARL3	DB00197	Troglitazone	-9.2
ARL3	DB00496	Darifenacin	-9.1
ARL3	DB08930	Dolutegravir	-9.1
ARL3	DB00246	Ziprasidone	-9.1
ARL3	DB11978	Glasdegib	-9.1
ARL3	DB01184	Domperidone	-9
ARL3	DB12867	Benperidol	-9

ARL3	DB00642	Pemetrexed	-9
ARL3	DB06814	Protokylol	-9
ARL3	DB04038	Ergosterol	-9
ARL3	DB00450	Droperidol	-8.9
ARL3	DB00179	Masoprocol	-8.9
ARL3	DB06603	Panobinostat	-8.9
ARL3	DB13954	Estradiol cypionate	-8.9
ARL3	DB05016	Ataluren	-8.9
ARL3	DB00650	Leucovorin	-8.9
ARL3	DB11596	Levoleucovorin	-8.9
ARL3	DB01259	Lapatinib	-8.8
ARL3	DB00875	Flupentixol	-8.8
ARL3	DB00734	Risperidone	-8.8
ARL3	DB00276	Amsacrine	-8.8
ARL3	DB08912	Dabrafenib	-8.8
ARL3	DB12978	Pexidartinib	-8.8
ARL3	DB00795	Sulfasalazine	-8.7
ARL3	DB08931	Riociguat	-8.7
ARL3	DB00563	Methotrexate	-8.7
ARL3	DB11614	Rupatadine	-8.7
ARL3	DB06237	Avanafil	-8.7
ARL3	DB01267	Paliperidone	-8.7
ARL3	DB01102	Arbutamine	-8.7

ARL3	DB08865	Crizotinib	-8.7
ARL3	DB01003	Cromoglicic acid	-8.7
ARL3	DB01624	Zuclopenthixol	-8.7
ARL3	DB06212	Tolvaptan	-8.6
ARL3	DB00984	Nandrolone phenpropionate	-8.6
ARL3	DB01012	Cinacalcet	-8.6
ARL3	DB12364	Betrixaban	-8.6
ARL3	DB01392	Yohimbine	-8.6
ARL3	DB08954	Ifenprodil	-8.6
ARL3	DB15035	Zanubrutinib	-8.6
ARL3	DB06684	Vilazodone	-8.6
ARL3	DB06202	Lasofoxifene	-8.6
ARL3	DB00568	Cinnarizine	-8.6
ARL3	DB00841	Dobutamine	-8.6
ARL3	DB00843	Donepezil	-8.5
ARL3	DB01609	Deferasirox	-8.5
ARL3	DB09038	Empagliflozin	-8.5
ARL3	DB04908	Flibanserin	-8.5
ARL3	DB01219	Dantrolene	-8.5
ARL3	DB08815	Lurasidone	-8.5
ARL3	DB00448	Lansoprazole	-8.5
ARL3	DB11730	Ribociclib	-8.5
ARL3	DB00384	Triamterene	-8.5

ARL3	DB00385	Valrubicin	-8.5
ARL3	DB00755	Tretinoin	-8.5
ARL3	DB01148	Flavoxate	-8.5
ARL3	DB00757	Dolasetron	-8.5
ARL3	DB00737	Meclizine	-8.5
ARL3	DB09128	Brexpiprazole	-8.5
ARL3	DB00808	Indapamide	-8.5
ARL3	DB00158	Folic acid	-8.4
ARL3	DB00820	Tadalafil	-8.4
ARL3	DB11632	Opicapone	-8.4
ARL3	DB12020	Tecovirimat	-8.4
ARL3	DB13074	Macimorelin	-8.4
ARL3	DB00805	Minaprine	-8.4
ARL3	DB09195	Lorpiprazole	-8.4
ARL3	DB11636	Nomegestrol	-8.4
ARL3	DB12095	Telotristat ethyl	-8.4
ARL3	DB01268	Sunitinib	-8.4
ARL3	DB00524	Metolazone	-8.4
ARL3	DB01091	Butenafine	-8.4
ARL3	DB09073	Palbociclib	-8.4
ARL3	DB01288	Fenoterol	-8.4
ARL3	DB13766	Lidoflazine	-8.4
ARL3	DB14914	Flortaucipir F-18	-8.4

ARL3	DB00471	Montelukast	-8.3
ARL3	DB14840	Ripretinib	-8.3
ARL3	DB00157	NADH	-8.3
ARL3	DB01436	Alfacalcidol	-8.3
ARL3	DB11703	Acalabrutinib	-8.3
ARL3	DB11760	Talazoparib	-8.3
ARL3	DB00637	Astemizole	-8.2
ARL3	DB00656	Trazodone	-8.2
ARL3	DB06290	Simeprevir	-8.1
ARL3	DB01232	Saquinavir	-8
ARL3	DB09272	Eluxadoline	-6.9
CNNM2	DB12457	Rimegepant	-10
CNNM2	DB01419	Antrafenine	-10
CNNM2	DB05039	Indacaterol	-9.9
CNNM2	DB11942	Selinexor	-9.9
CNNM2	DB11791	Capmatinib	-9.9
CNNM2	DB13931	Netarsudil	-9.9
CNNM2	DB09291	Rolapitant	-9.7
CNNM2	DB04861	Nebivolol	-9.7
CNNM2	DB00224	Indinavir	-9.7
CNNM2	DB12877	Oxatomide	-9.6
CNNM2	DB00157	NADH	-9.6
CNNM2	DB00549	Zafirlukast	-9.6

CNNM2	DB13074	Macimorelin	-9.5
CNNM2	DB00619	Imatinib	-9.5
CNNM2	DB01251	Gliquidone	-9.4
CNNM2	DB12867	Benperidol	-9.3
CNNM2	DB06608	Tafenoquine	-9.3
CNNM2	DB00564	Carbamazepine	-9.3
CNNM2	DB00266	Dicoumarol	-9.3
CNNM2	DB00973	Ezetimibe	-9.3
CNNM2	DB00878	Chlorhexidine	-9.3
CNNM2	DB09280	Lumacaftor	-9.3
CNNM2	DB06603	Panobinostat	-9.2
CNNM2	DB01016	Glyburide	-9.2
CNNM2	DB00358	Mefloquine	-9.2
CNNM2	DB06228	Rivaroxaban	-9.2
CNNM2	DB11943	Delafloxacin	-9.2
CNNM2	DB00932	Tipranavir	-9.2
CNNM2	DB04868	Nilotinib	-9.2
CNNM2	DB00450	Droperidol	-9.1
CNNM2	DB00875	Flupentixol	-9.1
CNNM2	DB04842	Fluspirilene	-9.1
CNNM2	DB12301	Doravirine	-9.1
CNNM2	DB01012	Cinacalcet	-9
CNNM2	DB01003	Cromoglicic acid	-9

CNNM2	DB04908	Flibanserin	-9
CNNM2	DB00776	Oxcarbazepine	-9
CNNM2	DB00751	Epinastine	-9
CNNM2	DB00525	Tolnaftate	-9
CNNM2	DB12332	Rucaparib	-9
CNNM2	DB08976	Floctafenine	-9
CNNM2	DB00222	Glimepiride	-9
CNNM2	DB00209	Trospium	-8.9
CNNM2	DB00598	Labetalol	-8.9
CNNM2	DB00434	Cyproheptadine	-8.9
CNNM2	DB01238	Aripiprazole	-8.9
CNNM2	DB08815	Lurasidone	-8.9
CNNM2	DB01261	Sitagliptin	-8.9
CNNM2	DB12978	Pexidartinib	-8.9
CNNM2	DB11963	Dacomitinib	-8.8
CNNM2	DB00342	Terfenadine	-8.8
CNNM2	DB00673	Aprepitant	-8.8
CNNM2	DB11732	Lasmiditan	-8.8
CNNM2	DB01624	Zuclopenthixol	-8.8
CNNM2	DB08907	Canagliflozin	-8.8
CNNM2	DB00303	Ertapenem	-8.8
CNNM2	DB01608	Periciazine	-8.8
CNNM2	DB00905	Bimatoprost	-8.8

CNNM2	DB00276	Amsacrine	-8.7
CNNM2	DB00298	Dapiprazole	-8.7
CNNM2	DB00737	Meclizine	-8.7
CNNM2	DB01091	Butenafine	-8.7
CNNM2	DB04825	Prenylamine	-8.7
CNNM2	DB09272	Eluxadoline	-8.7
CNNM2	DB00757	Dolasetron	-8.7
CNNM2	DB08954	Ifenprodil	-8.7
CNNM2	DB08950	Indoramin	-8.6
CNNM2	DB06684	Vilazodone	-8.6
CNNM2	DB00637	Astemizole	-8.6
CNNM2	DB00735	Naftifine	-8.6
CNNM2	DB00670	Pirenzepine	-8.6
CNNM2	DB06274	Alvimopan	-8.6
CNNM2	DB00605	Sulindac	-8.6
CNNM2	DB06814	Protokylol	-8.6
CNNM2	DB09319	Carindacillin	-8.6
CNNM2	DB01149	Nefazodone	-8.6
CNNM2	DB00192	Indecainide	-8.5
CNNM2	DB00831	Trifluoperazine	-8.5
CNNM2	DB00656	Trazodone	-8.5
CNNM2	DB04898	Ximelagatran	-8.5
CNNM2	DB00693	Fluorescein	-8.5

CNNM2	DB08930	Dolutegravir	-8.5
CNNM2	DB00354	Buclizine	-8.5
CNNM2	DB01184	Domperidone	-8.5
CNNM2	DB04841	Flunarizine	-8.5
CNNM2	DB00715	Paroxetine	-8.5
CNNM2	DB08896	Regorafenib	-8.5
CNNM2	DB01102	Arbutamine	-8.5
CNNM2	DB06813	Pralatrexate	-8.4
CNNM2	DB00363	Clozapine	-8.4
CNNM2	DB00377	Palonosetron	-8.4
CNNM2	DB00897	Triazolam	-8.4
CNNM2	DB11699	Tropisetron	-8.4
CNNM2	DB08860	Pitavastatin	-8.4
CNNM2	DB01418	Acenocoumarol	-8.4
CNNM2	DB00251	Terconazole	-8.4
CNNM2	DB00353	Methylergometrine	-8.3
CNNM2	DB00947	Fulvestrant	-8.3
CNNM2	DB05016	Ataluren	-8.3
CNNM2	DB06401	Bazedoxifene	-8.2

Reference

1 Adler, C. M., Levine, A. D., DelBello, M. P. & Strakowski, S. M. Changes in gray matter volume in patients with bipolar disorder. *Biological psychiatry* **58**, 151-157, doi:10.1016/j.biopsych.2005.03.022 (2005).

2 Alonso-Lana, S. *et al.* Structural and Functional Brain Correlates of Cognitive Impairment in Euthymic Patients with Bipolar Disorder. *PloS one* **11**, e0158867, doi:10.1371/journal.pone.0158867 (2016).

3 Altamura, A. C. *et al.* Structural and metabolic differentiation between bipolar disorder with psychosis and substance-induced psychosis: An integrated MRI/PET study. *European psychiatry : the journal of the Association of European Psychiatrists* **41**, 85-94, doi:10.1016/j.eurpsy.2016.09.009 (2017).

4 Ambrosi, E. *et al.* Structural brain alterations in bipolar disorder II: a combined voxel-based morphometry (VBM) and diffusion tensor imaging (DTI) study. *Journal of affective disorders* **150**, 610-615, doi:10.1016/j.jad.2013.02.023 (2013).

5 Baez, S. *et al.* Brain structural correlates of executive and social cognition profiles in behavioral variant frontotemporal dementia and elderly bipolar disorder. *Neuropsychologia* **126**, 159-169, doi:10.1016/j.neuropsychologia.2017.02.012 (2019).

6 Brown, G. G. *et al.* Voxel-based morphometry of patients with schizophrenia or bipolar I disorder: a matched control study. *Psychiatry research* **194**, 149-156, doi:10.1016/j.pscychresns.2011.05.005 (2011).

7 Bruno, S. D., Barker, G. J., Cercignani, M., Symms, M. & Ron, M. A. A study of bipolar disorder using magnetization transfer imaging and voxel-based morphometry. *Brain : a journal of neurology* **127**, 2433-2440, doi:10.1093/brain/awh274 (2004).

8 Cai, Y. *et al.* Grey matter volume abnormalities in patients with bipolar I depressive disorder and unipolar depressive disorder: a voxel-based

morphometry study. *Neuroscience bulletin* **31**, 4-12, doi:10.1007/s12264-014-1485-5 (2015).

9 Chen, X., Wen, W., Malhi, G. S., Ivanovski, B. & Sachdev, P. S. Regional gray matter changes in bipolar disorder: a voxel-based morphometric study. *The Australian and New Zealand journal of psychiatry* **41**, 327-336, doi:10.1080/00048670701213229 (2007).

10 Chen, L. *et al.* Common and distinct abnormal frontal-limbic system structural and functional patterns in patients with major depression and bipolar disorder. *NeuroImage. Clinical* **20**, 42-50, doi:10.1016/j.nicl.2018.07.002 (2018).

11 Eker, C. *et al.* Brain regions associated with risk and resistance for bipolar I disorder: a voxel-based MRI study of patients with bipolar disorder and their healthy siblings. *Bipolar disorders* **16**, 249-261, doi:10.1111/bdi.12181 (2014).

12 Emsell, L. *et al.* White matter differences in euthymic bipolar I disorder: a combined magnetic resonance imaging and diffusion tensor imaging voxel-based study. *Bipolar disorders* **15**, 365-376, doi:10.1111/bdi.12073 (2013).

13 Frangou, S. Brain structural and functional correlates of resilience to Bipolar Disorder. *Frontiers in human neuroscience* **5**, 184, doi:10.3389/fnhum.2011.00184 (2011).

14 Goikolea, J. M. *et al.* Multimodal Brain Changes in First-Episode Mania: A Voxel-Based Morphometry, Functional Magnetic Resonance Imaging, and Connectivity Study. *Schizophrenia bulletin* **45**, 464-473, doi:10.1093/schbul/sby047 (2019).

15 Hajek, T. *et al.* Insulin resistance, diabetes mellitus, and brain structure in bipolar disorders. *Neuropsychopharmacology : official publication of the American College of Neuropsychopharmacology* **39**, 2910-2918, doi:10.1038/npp.2014.148 (2014).

16 Ivleva, E. I. *et al.* Gray matter volume as an intermediate phenotype for

psychosis: Bipolar-Schizophrenia Network on Intermediate Phenotypes (B-SNIP). *The American journal of psychiatry* **170**, 1285-1296, doi:10.1176/appi.ajp.2013.13010126 (2013).

17 Kandilarova, S., Stoyanov, D., Sirakov, N., Maes, M. & Specht, K. Reduced grey matter volume in frontal and temporal areas in depression: contributions from voxel-based morphometry study. *Acta neuropsychiatrica* **31**, 252-257, doi:10.1017/neu.2019.20 (2019).

18 Lee, D.-K. *et al.* Common gray and white matter abnormalities in schizophrenia and bipolar disorder. **15**, e0232826 (2020).

19 Li, M. *et al.* Voxel-based morphometric analysis on the volume of gray matter in bipolar I disorder. *Psychiatry research* **191**, 92-97, doi:10.1016/j.pscychresns.2010.09.006 (2011).

20 Lochhead, R. A., Parsey, R. V., Oquendo, M. A. & Mann, J. J. Regional brain gray matter volume differences in patients with bipolar disorder as assessed by optimized voxel-based morphometry. *Biological psychiatry* **55**, 1154-1162, doi:10.1016/j.biopsych.2004.02.026 (2004).

21 McDonald, C. *et al.* Regional volume deviations of brain structure in schizophrenia and psychotic bipolar disorder: computational morphometry study. *The British journal of psychiatry : the journal of mental science* **186**, 369-377, doi:10.1192/bjp.186.5.369 (2005).

22 Metin, B., Farhad, S., Erguzel, T., Çiftçi, E. & Tarhan, N. Combined use of gray matter volume and neuropsychological test performance for classification of individuals with bipolar I disorder via artificial neural network method. *Journal of neural transmission (Vienna, Austria : 1996)* **130**, 967-974, doi:10.1007/s00702-023-02649-y (2023).

23 Miola, A. *et al.* Gray matter volume covariance networks are associated with altered emotional processing in bipolar disorder: a source-based morphometry study. *Brain imaging and behavior* **16**, 738-747, doi:10.1007/s11682-021-00541-5 (2022).

24 Molina, V. *et al.* Different gray matter patterns in chronic schizophrenia and

chronic bipolar disorder patients identified using voxel-based morphometry. *European archives of psychiatry and clinical neuroscience* **261**, 313-322, doi:10.1007/s00406-010-0183-1 (2011).

25 Narita, K. *et al.* Volume reduction of ventromedial prefrontal cortex in bipolar II patients with rapid cycling: a voxel-based morphometric study. *Progress in neuro-psychopharmacology & biological psychiatry* **35**, 439-445, doi:10.1016/j.pnpbp.2010.11.030 (2011).

26 Nenadic, I. *et al.* Brain structure in schizophrenia vs. psychotic bipolar I disorder: A VBM study. *Schizophrenia research* **165**, 212-219, doi:10.1016/j.schres.2015.04.007 (2015).

27 Nery, F. G. *et al.* Gray matter volumes in patients with bipolar disorder and their first-degree relatives. *Psychiatry research* **234**, 188-193, doi:10.1016/j.pscychresns.2015.09.005 (2015).

28 Neves Mde, C. *et al.* A voxel-based morphometry study of gray matter correlates of facial emotion recognition in bipolar disorder. *Psychiatry research* **233**, 158-164, doi:10.1016/j.pscychresns.2015.05.009 (2015).

29 Nugent, A. C. *et al.* Cortical abnormalities in bipolar disorder investigated with MRI and voxel-based morphometry. *NeuroImage* **30**, 485-497, doi:10.1016/j.neuroimage.2005.09.029 (2006).

30 Ota, M. *et al.* Effects of ankyrin 3 gene risk variants on brain structures in patients with bipolar disorder and healthy subjects. *Psychiatry and clinical neurosciences* **70**, 498-506, doi:10.1111/pcn.12431 (2016).

31 Poletti, S. *et al.* Adverse childhood experiences influence the detrimental effect of bipolar disorder and schizophrenia on cortico-limbic grey matter volumes. *Journal of affective disorders* **189**, 290-297, doi:10.1016/j.jad.2015.09.049 (2016).

32 Quidé, Y., Wilhelmi, C. & Green, M. J. Structural brain morphometry associated with theory of mind in bipolar disorder and schizophrenia. *PsyCh journal* **9**, 234-246, doi:10.1002/pchj.322 (2020).

33 Rocha-Rego, V. *et al.* Examination of the predictive value of structural

magnetic resonance scans in bipolar disorder: a pattern classification approach. *Psychological medicine* **44**, 519-532, doi:10.1017/s0033291713001013 (2014).

34 Sani, G. *et al.* Gray and white matter trajectories in patients with bipolar disorder. *Bipolar disorders* **18**, 52-62, doi:10.1111/bdi.12359 (2016).

35 Sarıçiçek, A. *et al.* Neuroanatomical correlates of genetic risk for bipolar disorder: A voxel-based morphometry study in bipolar type I patients and healthy first degree relatives. *Journal of affective disorders* **186**, 110-118, doi:10.1016/j.jad.2015.06.055 (2015).

36 Scherk, H. *et al.* No change to grey and white matter volumes in bipolar I disorder patients. *European archives of psychiatry and clinical neuroscience* **258**, 345-349, doi:10.1007/s00406-007-0801-8 (2008).

37 Song, J. *et al.* Differences in gray matter volume corresponding to delusion and hallucination in patients with schizophrenia compared with patients who have bipolar disorder. *Neuropsychiatric disease and treatment* **11**, 1211-1219, doi:10.2147/ndt.S80438 (2015).

38 Song, H. *et al.* Cortical Volumetric Correlates of Childhood Trauma, Anxiety, and Impulsivity in Bipolar Disorder. *Psychiatry investigation* **17**, 627-635, doi:10.30773/pi.2019.0305 (2020).

39 Sun, N. *et al.* Fractional amplitude of low-frequency fluctuations and gray matter volume alterations in patients with bipolar depression. *Neuroscience letters* **730**, 135030, doi:10.1016/j.neulet.2020.135030 (2020).

40 Tang, L. R. *et al.* Voxel-based morphometry study of the insular cortex in bipolar depression. *Psychiatry research* **224**, 89-95, doi:10.1016/j.pscychresns.2014.08.004 (2014).

41 Thomas-Odenthal, F. *et al.* Larger putamen in individuals at risk and with manifest bipolar disorder. *Psychological medicine* **54**, 3071-3081, doi:10.1017/s0033291724001193 (2024).

42 Thiel, K. *et al.* White and gray matter alterations in bipolar I and bipolar II disorder subtypes compared with healthy controls - exploring associations

with disease course and polygenic risk. *Neuropsychopharmacology : official publication of the American College of Neuropsychopharmacology* **49**, 814-823, doi:10.1038/s41386-024-01812-7 (2024).

43 Watson, D. R. *et al.* A voxel based morphometry study investigating brain structural changes in first episode psychosis. *Behavioural brain research* **227**, 91-99, doi:10.1016/j.bbr.2011.10.034 (2012).

44 Yang, Y. *et al.* Reduced Gray Matter Volume in Orbitofrontal Cortex Across Schizophrenia, Major Depressive Disorder, and Bipolar Disorder: A Comparative Imaging Study. *Frontiers in neuroscience* **16**, 919272, doi:10.3389/fnins.2022.919272 (2022).

45 Yip, S. W., Chandler, R. A., Rogers, R. D., Mackay, C. E. & Goodwin, G. M. White matter alterations in antipsychotic- and mood stabilizer-naïve individuals with bipolar II/NOS disorder. *NeuroImage. Clinical* **3**, 271-278, doi:10.1016/j.nicl.2013.08.005 (2013).

46 Yüksel, C. *et al.* Gray matter volume in schizophrenia and bipolar disorder with psychotic features. *Schizophrenia research* **138**, 177-182, doi:10.1016/j.schres.2012.03.003 (2012).

47 Zhang, Y.-N. *et al.* Healthy individuals vs patients with bipolar or unipolar depression in gray matter volume. **9**, 1304 (2021).

48 Adamu, M. J. *et al.* Unraveling the pathophysiology of schizophrenia: insights from structural magnetic resonance imaging studies. *Frontiers in psychiatry* **14**, 1188603, doi:10.3389/fpsyt.2023.1188603 (2023).

49 Ananth, H. *et al.* Cortical and subcortical gray matter abnormalities in schizophrenia determined through structural magnetic resonance imaging with optimized volumetric voxel-based morphometry. *The American journal of psychiatry* **159**, 1497-1505, doi:10.1176/appi.ajp.159.9.1497 (2002).

50 Anderson, V. M., Goldstein, M. E., Kydd, R. R. & Russell, B. R. Extensive gray matter volume reduction in treatment-resistant schizophrenia. *The international journal of neuropsychopharmacology* **18**, pyv016, doi:10.1093/ijnp/pyv016 (2015).

51 Antonova, E. *et al.* The relationship of structural alterations to cognitive deficits in schizophrenia: a voxel-based morphometry study. **58**, 457-467 (2005).

52 Asami, T. *et al.* Longitudinal loss of gray matter volume in patients with first-episode schizophrenia: DARTEL automated analysis and ROI validation. *NeuroImage* **59**, 986-996, doi:10.1016/j.neuroimage.2011.08.066 (2012).

53 Bagary, M. S. *et al.* Gray and white matter brain abnormalities in first-episode schizophrenia inferred from magnetization transfer imaging. **60**, 779-788 (2003).

54 Bergé, D. *et al.* Gray matter volume deficits and correlation with insight and negative symptoms in first-psychotic-episode subjects. *Acta psychiatrica Scandinavica* **123**, 431-439, doi:10.1111/j.1600-0447.2010.01635.x (2011).

55 Biondi, M., Marino, M., Mantini, D. & Spironelli, C. Brain Structural Alterations Underlying Mood-Related Deficits in Schizophrenia. *Biomedicines* **13**, doi:10.3390/biomedicines13030736 (2025).

56 Bonilha, L. *et al.* Neurocognitive deficits and prefrontal cortical atrophy in patients with schizophrenia. *Schizophrenia research* **101**, 142-151, doi:10.1016/j.schres.2007.11.023 (2008).

57 Borgwardt, S. J. *et al.* Regional gray matter volume in monozygotic twins concordant and discordant for schizophrenia. **67**, 956-964 (2010).

58 Bose, S. K. *et al.* The effect of ageing on grey and white matter reductions in schizophrenia. *Schizophrenia research* **112**, 7-13, doi:10.1016/j.schres.2009.04.023 (2009).

59 Cascella, N. G. *et al.* Gray-matter abnormalities in deficit schizophrenia. *Schizophrenia research* **120**, 63-70, doi:10.1016/j.schres.2010.03.039 (2010).

60 Chow, E. W. *et al.* Association of schizophrenia in 22q11.2 deletion syndrome and gray matter volumetric deficits in the superior temporal gyrus. *The American journal of psychiatry* **168**, 522-529, doi:10.1176/appi.ajp.2010.10081230 (2011).

61 Cooke, M. A. *et al.* Neurological basis of poor insight in psychosis: a

voxel-based MRI study. *Schizophrenia research* **103**, 40-51, doi:10.1016/j.schres.2008.04.022 (2008).

62 Ellison-Wright, I. *et al.* Distribution of tract deficits in schizophrenia. *BMC psychiatry* **14**, 99, doi:10.1186/1471-244x-14-99 (2014).

63 Euler, M., Thoma, R. J., Gangestad, S. W., Cañive, J. M. & Yeo, R. A. The impact of developmental instability on Voxel-Based Morphometry analyses of neuroanatomical abnormalities in schizophrenia. *Schizophrenia research* **115**, 1-7, doi:10.1016/j.schres.2009.08.014 (2009).

64 Ferri, F. *et al.* Action verb understanding in first-episode schizophrenia: is there evidence for a simulation deficit? *Neuropsychologia* **50**, 988-996, doi:10.1016/j.neuropsychologia.2012.02.005 (2012).

65 Filippi, M. *et al.* Patterns of brain structural changes in first-contact, antipsychotic drug-naive patients with schizophrenia. **35**, 30-37 (2014).

66 Frascarelli, M. *et al.* Medial frontal gyrus alterations in schizophrenia: relationship with duration of illness and executive dysfunction. *Psychiatry research* **231**, 103-110, doi:10.1016/j.pscychresns.2014.10.017 (2015).

67 Fukuta, H. *et al.* Effects of menopause on brain structural changes in schizophrenia. *Psychiatry and clinical neurosciences* **67**, 3-11, doi:10.1111/pcn.12003 (2013).

68 Garcia-Marti, G. *et al.* Progressive loss of cortical gray matter in first episode psychosis patients with auditory hallucinations. *Schizophrenia research* **267**, 534-545, doi:10.1016/j.schres.2023.11.011 (2024).

69 Guo, X. *et al.* Duration of untreated psychosis is associated with temporal and occipitotemporal gray matter volume decrease in treatment naive schizophrenia. **8**, e83679 (2013).

70 Gou, N. *et al.* Structural Deficits in the Frontotemporal Network Associated With Psychopathic Traits in Violent Offenders With Schizophrenia. *Frontiers in psychiatry* **13**, 846838, doi:10.3389/fpsyt.2022.846838 (2022).

71 Honea, R. A. *et al.* Is gray matter volume an intermediate phenotype for schizophrenia? A voxel-based morphometry study of patients with

schizophrenia and their healthy siblings. **63**, 465-474 (2008).

72 Hooker, C. I., Bruce, L., Lincoln, S. H., Fisher, M. & Vinogradov, S. J. B. p. Theory of mind skills are related to gray matter volume in the ventromedial prefrontal cortex in schizophrenia. **70**, 1169-1178 (2011).

73 Horacek, J. *et al.* Latent toxoplasmosis reduces gray matter density in schizophrenia but not in controls: voxel-based-morphometry (VBM) study. *The world journal of biological psychiatry : the official journal of the World Federation of Societies of Biological Psychiatry* **13**, 501-509, doi:10.3109/15622975.2011.573809 (2012).

74 Horn, H. *et al.* Gray matter volume differences specific to formal thought disorder in schizophrenia. *Psychiatry research* **182**, 183-186, doi:10.1016/j.pscychresns.2010.01.016 (2010).

75 Huang, P. *et al.* Decreased bilateral thalamic gray matter volume in first-episode schizophrenia with prominent hallucinatory symptoms: A volumetric MRI study. *Scientific reports* **5**, 14505, doi:10.1038/srep14505 (2015).

76 Huang, X. *et al.* Decreased Left Putamen and Thalamus Volume Correlates with Delusions in First-Episode Schizophrenia Patients. *Frontiers in psychiatry* **8**, 245, doi:10.3389/fpsyt.2017.00245 (2017).

77 Hýža, M., Huttlová, J., Keřkovský, M. & Kašpárek, T. Psychosis effect on hippocampal reduction in schizophrenia. *Progress in neuro-psychopharmacology & biological psychiatry* **48**, 186-192, doi:10.1016/j.pnpbp.2013.10.008 (2014).

78 Jayakumar, P. N., Venkatasubramanian, G., Gangadhar, B. N., Janakiramaiah, N. & Keshavan, M. S. Optimized voxel-based morphometry of gray matter volume in first-episode, antipsychotic-naive schizophrenia. *Progress in neuro-psychopharmacology & biological psychiatry* **29**, 587-591, doi:10.1016/j.pnpbp.2005.01.020 (2005).

79 Kaspárek, T. *et al.* Prefrontal but not temporal grey matter changes in males with first-episode schizophrenia. *Progress in neuro-psychopharmacology &*

biological psychiatry **31**, 151-157, doi:10.1016/j.pnpbp.2006.08.011 (2007).

80 Katz, J. *et al.* Similar white matter but opposite grey matter changes in schizophrenia and high-functioning autism. **134**, 31-39 (2016).

81 Kim, G. W., Kim, Y. H. & Jeong, G. W. Whole brain volume changes and its correlation with clinical symptom severity in patients with schizophrenia: A DARTEL-based VBM study. *PloS one* **12**, e0177251, doi:10.1371/journal.pone.0177251 (2017).

82 Koelkebeck, K. *et al.* Gray matter volume reductions in patients with schizophrenia: A replication study across two cultural backgrounds. *Psychiatry research. Neuroimaging* **292**, 32-40, doi:10.1016/j.pscychresns.2019.08.008 (2019).

83 Kong, L. *et al.* Comparison of grey matter volume and thickness for analysing cortical changes in chronic schizophrenia: a matter of surface area, grey/white matter intensity contrast, and curvature. *Psychiatry research* **231**, 176-183, doi:10.1016/j.pscychresns.2014.12.004 (2015).

84 Li, C. *et al.* Voxel-based morphometry results in first-episode schizophrenia: a comparison of publicly available software packages. *Brain imaging and behavior* **14**, 2224-2231, doi:10.1007/s11682-019-00172-x (2020).

85 Liao, J. *et al.* Reduced paralimbic system gray matter volume in schizophrenia: Correlations with clinical variables, symptomatology and cognitive function. *Journal of psychiatric research* **65**, 80-86, doi:10.1016/j.jpsychires.2015.04.008 (2015).

86 Lu, J. *et al.* Brain structural alterations associated with impulsiveness in male violent patients with schizophrenia. *BMC psychiatry* **24**, 281, doi:10.1186/s12888-024-05721-3 (2024).

87 Ma, M. *et al.* Common and Distinct Alterations of Cognitive Function and Brain Structure in Schizophrenia and Major Depressive Disorder: A Pilot Study. *Frontiers in psychiatry* **12**, 705998, doi:10.3389/fpsyt.2021.705998 (2021).

88 Maggioni, E. *et al.* Common and distinct structural features of schizophrenia

and bipolar disorder: The European Network on Psychosis, Affective disorders and Cognitive Trajectory (ENPACT) study. *PloS one* **12**, e0188000, doi:10.1371/journal.pone.0188000 (2017).

89 Nakamura, K. *et al.* Gray matter changes in subjects at high risk for developing psychosis and first-episode schizophrenia: a voxel-based structural MRI study. *Frontiers in psychiatry* **4**, 16, doi:10.3389/fpsyt.2013.00016 (2013).

90 Nemoto, K. *et al.* Differentiation of schizophrenia using structural MRI with consideration of scanner differences: A real-world multisite study. *Psychiatry and clinical neurosciences* **74**, 56-63, doi:10.1111/pcn.12934 (2020).

91 Neugebauer, K. *et al.* Nerve Growth Factor Serum Levels Are Associated With Regional Gray Matter Volume Differences in Schizophrenia Patients. *Frontiers in psychiatry* **10**, 275, doi:10.3389/fpsyt.2019.00275 (2019).

92 Oertel-Knöchel, V. *et al.* Cortical-basal ganglia imbalance in schizophrenia patients and unaffected first-degree relatives. *Schizophrenia research* **138**, 120-127, doi:10.1016/j.schres.2012.02.029 (2012).

93 Onay, A., Yapıcı Eser, H., Ulaşoğlu Yıldız, Ç., Aslan, S. & Talı, E. T. A combined VBM and DTI study of schizophrenia: bilateral decreased insula volume and cerebral white matter disintegrity corresponding to subinsular white matter projections unlinked to clinical symptomatology. *Diagnostic and interventional radiology (Ankara, Turkey)* **23**, 390-397, doi:10.5152/dir.2017.16519 (2017).

94 Ota, M. *et al.* Correlation of reduced social communicational and interactional skills with regional grey matter volumes in schizophrenia patients. *Acta neuropsychiatrica* **29**, 374-381, doi:10.1017/neu.2017.9 (2017).

95 Palaniyappan, L. & Liddle, P. F. Differential effects of surface area, gyrification and cortical thickness on voxel based morphometric deficits in schizophrenia. *NeuroImage* **60**, 693-699, doi:10.1016/j.neuroimage.2011.12.058 (2012).

96 Picado, M. *et al.* The neuroanatomical basis of panic disorder and social phobia in schizophrenia: a voxel based morphometric study. *PloS one* **10**, e0119847, doi:10.1371/journal.pone.0119847 (2015).

97 Ren, W. *et al.* Anatomical and functional brain abnormalities in drug-naive first-episode schizophrenia. *The American journal of psychiatry* **170**, 1308-1316, doi:10.1176/appi.ajp.2013.12091148 (2013).

98 Rigucci, S. *et al.* Anatomical substrates of cognitive and clinical dimensions in first episode schizophrenia. **128**, 261-270 (2013).

99 Rootes-Murdy, K., Zendehrouh, E., Calhoun, V. D. & Turner, J. A. Spatially Covarying Patterns of Gray Matter Volume and Concentration Highlight Distinct Regions in Schizophrenia. *Frontiers in neuroscience* **15**, 708387, doi:10.3389/fnins.2021.708387 (2021).

100 Rose, E. J. *et al.* The miR-137 schizophrenia susceptibility variant rs1625579 does not predict variability in brain volume in a sample of schizophrenic patients and healthy individuals. *American journal of medical genetics. Part B, Neuropsychiatric genetics : the official publication of the International Society of Psychiatric Genetics* **165b**, 467-471, doi:10.1002/ajmg.b.32249 (2014).

101 Ružić Baršić, A. *et al.* Onset of Schizophrenia Prior to the End of Brain Maturation Alters Grey Matter Volume Loss. *Psychiatria Danubina* **33**, 719-731 (2021).

102 Salgado-Pineda, P. *et al.* Decreased cerebral activation during CPT performance: structural and functional deficits in schizophrenic patients. *NeuroImage* **21**, 840-847, doi:10.1016/j.neuroimage.2003.10.027 (2004).

103 Salgado-Pineda, P. *et al.* Correlated structural and functional brain abnormalities in the default mode network in schizophrenia patients. *Schizophrenia research* **125**, 101-109, doi:10.1016/j.schres.2010.10.027 (2011).

104 Sanjuán, J. *et al.* FOXP2 expression and gray matter density in the male brains of patients with schizophrenia. *Brain imaging and behavior* **15**,

1403-1411, doi:10.1007/s11682-020-00339-x (2021).

105 Sarro, S. *et al.* Structural brain changes associated with tardive dyskinesia in schizophrenia. **203**, 51-57 (2013).

106 Schiffer, B. *et al.* Structural brain alterations associated with schizophrenia preceded by conduct disorder: a common and distinct subtype of schizophrenia? *Schizophrenia bulletin* **39**, 1115-1128, doi:10.1093/schbul/sbs115 (2013).

107 Schuster, C. *et al.* Gray matter volume decreases in elderly patients with schizophrenia: a voxel-based morphometry study. *Schizophrenia bulletin* **38**, 796-802, doi:10.1093/schbul/sbq150 (2012).

108 Siddi, S. *et al.* Depression, auditory-verbal hallucinations, and delusions in patients with schizophrenia: Different patterns of association with prefrontal gray and white matter volume. *Psychiatry research. Neuroimaging* **283**, 55-63, doi:10.1016/j.pscychresns.2018.12.001 (2019).

109 Singh, S. *et al.* Evidence for regional hippocampal damage in patients with schizophrenia. *Neuroradiology* **60**, 199-205, doi:10.1007/s00234-017-1954-4 (2018).

110 Stegmayer, K. *et al.* Structural brain correlates of defective gesture performance in schizophrenia. *Cortex; a journal devoted to the study of the nervous system and behavior* **78**, 125-137, doi:10.1016/j.cortex.2016.02.014 (2016).

111 Tan, S. *et al.* Brain Correlates of Self-Evaluation Deficits in Schizophrenia: A Combined Functional and Structural MRI Study. *PloS one* **10**, e0138737, doi:10.1371/journal.pone.0138737 (2015).

112 Tikàsz, A. *et al.* Reward-related decision-making in schizophrenia: A multimodal neuroimaging study. *Psychiatry research. Neuroimaging* **286**, 45-52, doi:10.1016/j.pscychresns.2019.03.007 (2019).

113 Tomelleri, L. *et al.* Brain structural changes associated with chronicity and antipsychotic treatment in schizophrenia. *European neuropsychopharmacology : the journal of the European College of*

Neuropsychopharmacology **19**, 835-840, doi:10.1016/j.euroneuro.2009.07.007 (2009).

114 Torres, U. S. *et al.* Patterns of regional gray matter loss at different stages of schizophrenia: A multisite, cross-sectional VBM study in first-episode and chronic illness. *NeuroImage. Clinical* **12**, 1-15, doi:10.1016/j.nicl.2016.06.002 (2016).

115 Tregellas, J. R. *et al.* Gray matter volume differences and the effects of smoking on gray matter in schizophrenia. *Schizophrenia research* **97**, 242-249, doi:10.1016/j.schres.2007.08.019 (2007).

116 Tseng, H. H. *et al.* Absence of negative associations of insular and medial frontal gray matter volume with dissociative symptoms in schizophrenia. *Journal of psychiatric research* **138**, 485-491, doi:10.1016/j.jpsychires.2021.04.017 (2021).

117 Van Assche, L. *et al.* A voxel- and source-based morphometry analysis of grey matter volume differences in very-late-onset schizophrenia-like psychosis. *Psychological medicine* **54**, 592-600, doi:10.1017/s0033291723002258 (2024).

118 van Tol, M. J. *et al.* Voxel-based gray and white matter morphometry correlates of hallucinations in schizophrenia: The superior temporal gyrus does not stand alone. *NeuroImage. Clinical* **4**, 249-257, doi:10.1016/j.nicl.2013.12.008 (2014).

119 Walther, S. *et al.* Structural alterations of the motor cortex and higher order cortical areas suggest early neurodevelopmental origin of catatonia in schizophrenia. *Schizophrenia research* **263**, 131-138, doi:10.1016/j.schres.2022.10.004 (2024).

120 Wu, C. *et al.* Cortical Gray Matter Loss, Augmented Vulnerability to Speech-on-Speech Masking, and Delusion in People With Schizophrenia. *Frontiers in psychiatry* **9**, 287, doi:10.3389/fpsyt.2018.00287 (2018).

121 Wu, H. *et al.* Gray matter reduction in bilateral insula mediating adverse psychiatric effects of body mass index in schizophrenia. *BMC*

psychiatry **22**, 639, doi:10.1186/s12888-022-04285-4 (2022).

122 Xie, Y. *et al.* rTMS Induces Brain Functional and Structural Alternations in Schizophrenia Patient With Auditory Verbal Hallucination. *Frontiers in neuroscience* **15**, 722894, doi:10.3389/fnins.2021.722894 (2021).

123 Yang, Z. Y. *et al.* Neural correlates of prospection impairments in schizophrenia: Evidence from voxel-based morphometry analysis. *Psychiatry research. Neuroimaging* **293**, 110987, doi:10.1016/j.pscychresns.2019.110987 (2019).

124 Yang, K. *et al.* Magnetic Resonance Imaging Characteristics of Brain Structure and Neuroendocrine Changes in Patients with First-Episode Schizophrenia. **2023** (2023).

125 Zhang, C. *et al.* Differential Cortical Gray Matter Deficits in Adolescent- and Adult-Onset First-Episode Treatment-Naïve Patients with Schizophrenia. *Scientific reports* **7**, 10267, doi:10.1038/s41598-017-10688-1 (2017).
